# Bioactive Profile, Antioxidant, and *In Vitro* Antimicrobial Activity of ‘Mortiño’ (*Vaccinium meridionale*) and Other *Rubus* and *Vaccinium* Andean Red Fruits at Different Stages of Ripeness

**DOI:** 10.3390/antiox15091091

**Published:** 2026-08-31

**Authors:** Elena Coyago-Cruz, Gabriela Méndez, Ruth Escobar-Quiñonez, Gabriela Cisneros, Jorge Heredia-Moya

**Affiliations:** 1 Carrera de Ingeniería en Biotecnología, Universidad Politécnica Salesiana, Sede Quito, Campus El Girón, Av. 12 de Octubre N2422 y Wilson, Quito 170143, Ecuador; 2 Maestría en Ingeniería Biomédica, Universidad Politécnica Salesiana, Sede Quito, Campus El Girón, Av. 12 de Octubre N2422 y Wilson, Quito 170143, Ecuador; 3 Centro de Investigación Biomédica (CENBIO), Facultad de Ciencias de la Salud Eugenio Espejo, Universidad UTE, Quito 170527, Ecuador

**Keywords:** DPPH, ABTS, microextraction, ultrasonic, RRLC, antibacterial activity, antifungal activity

## Abstract

This study evaluated changes in physicochemical characteristics, bioactive compounds, in vitro antioxidant activity, and antimicrobial activity during four ripening stages (M0%, M50%, M80%, and M100%) of ‘Castilla’ blackberry, ‘Buckingham Tayberry’ raspberry, ‘Heritage’ raspberry, blueberry, and ‘Mortiño’. Bioactive compounds were quantified using rapid-resolution liquid chromatography, antioxidant activity was determined using the ABTS and DPPH assays, and antimicrobial activity was evaluated through minimum inhibitory concentration assays against 13 bacteria and 7 fungal strains. Ripening effects were fruit- and compound-dependent. Soluble solids generally increased, and titratable acidity decreased during ripening. Early stages were characterised by higher concentrations of several organic and phenolic acids and, in some fruits, greater radical scavenging and antimicrobial responses. Advanced ripening favoured the accumulation of carotenoids and anthocyanins, particularly in blueberry and ‘Mortiño’. Antimicrobial activity also varies according to fruit type, ripening stage, and microorganism. ‘Mortiño’ showed the lowest MIC against *Enterococcus faecalis*, whereas the other fruits displayed selective inhibition against different bacterial and fungal strains. Overall, no single ripening stage maximised all compositional and biological variables, demonstrating that harvest stage selection should be fruit- and purpose-specific.

## 1. Introduction

In recent decades, growing interest in health and nutrition has increased the consumption of fruits and vegetables as dietary sources of bioactive compounds. Among these foods, berries, particularly those belonging to the Rosaceae and Ericaceae families, are valued for their sensory characteristics, nutritional composition, and potential health benefits [1]. Regular berry consumption has been associated with a lower risk of metabolic and cardiovascular diseases, mainly because these fruits contain vitamins, minerals, dietary fibre, and different classes of phytochemicals [2,3,4]. Their characteristic red, blue, and purple colours are primarily associated with anthocyanins and other pigments, while their bioactive composition also includes flavonoids, phenolic acids, carotenoids, and vitamin C [5,6]. Nevertheless, the concentrations of these compounds vary according to species, cultivar, genotype, agronomic management, environmental conditions, ripening stage, storage, and processing [1].

Among the berries cultivated or traditionally consumed in the Andean region, ‘Mortiño’ (*Vaccinium meridionale* Sw.) is a native species of Colombia and Ecuador. Its fruits are traditionally used in different preparations, including *colada morada*, an emblematic Ecuadorian beverage consumed during All Souls’ Day. ‘Mortiño’ contains sugars, minerals, B vitamins, vitamin C, and phenolic compounds, particularly anthocyanins [7,8,9]. ‘Castilla’ blackberry (*Rubus glaucus* Benth.) is also native to the South American Andes and is widely cultivated in Ecuador, Colombia, and Costa Rica. Fruits of the genus *Rubus* are recognised for their diverse phytochemical profiles and antioxidant properties, which may vary substantially among species, cultivars, and growing environments [10].

Other commercially important berries include blueberry (*Vaccinium corymbosum*), red raspberry (*Rubus idaeus*), and interspecific *Rubus* hybrids, such as ‘Buckingham Tayberry’. Blueberries contain anthocyanins, phenolic acids, flavonoids, tannins, vitamins, and minerals [11,12]. Red raspberries are characterised by their contents of sugars, organic acids, fibre, vitamins, anthocyanins, and ellagitannins [13]. Ripening is a coordinated physiological process involving changes in fruit colour, size, texture, soluble solids, acidity, and chemical composition. These transformations determine not only sensory and commercial quality but also the concentration and distribution of bioactive compounds. However, ripening-related changes do not follow a uniform pattern across berry species or compound classes. In Andean blackberry, early ripening stages have been associated with higher concentrations of polyphenols and flavonoids and greater antioxidant capacity, whereas anthocyanins become predominant in fully ripe fruits [14].

The bioactive composition of berries is relevant because phenolic compounds, carotenoids, vitamin C, and other reducing substances contribute to their *in vitro* antioxidant capacity [5,15,16]. Berry extracts have also shown antimicrobial activity against different bacterial and fungal strains [17,18,19].

Despite the extensive literature on the chemical composition and biological properties of berries, ripening-related changes are not uniform across fruit species, cultivars, or groups of compounds. Comparisons among studies are also limited by differences in maturity criteria, extraction procedures, analytical methods, and the expression of results. Consequently, a standardised comparison of physicochemical characteristics, individually quantified bioactive compounds, chemical antioxidant capacity, and antimicrobial responses across equivalent ripening stages may help clarify whether similar berry fruits follow common or species-specific ripening patterns. Moreover, few studies have simultaneously examined physicochemical characteristics, mineral composition, individually quantified bioactive compounds, *in vitro* antioxidant capacity, and antimicrobial activity throughout ripening.

In this context, it was hypothesised that ripening would produce fruit- and compound-dependent changes and that no single ripening stage would maximise all compositional and biological characteristics. Thus, the present study compared ‘Castilla’ blackberry, ‘Buckingham Tayberry’ raspberry, ‘Heritage’ raspberry, blueberry, and ‘Mortiño’ at four ripening stages (M0%, M50%, M80%, and M100%). Its specific contribution lies in the simultaneous evaluation of physicochemical characteristics, mineral composition, individually quantified bioactive compounds, *in vitro* antioxidant capacity, and antimicrobial activity against 13 bacterial and 7 fungal strains. The objective was to evaluate changes in their physicochemical characteristics, mineral composition, vitamin C, organic acids, carotenoids, chlorophylls, and their derivatives, phenolic compounds, *in vitro* antioxidant capacity, and antimicrobial activity. Rather than assuming that full maturity represents the optimal stage for all fruits and variables, this study examined whether the chemical and biological responses to ripening were fruit- and compound-dependent.

## 2. Materials and Methods

### 2.1. Physicochemical Analyses

**Figure 1:**
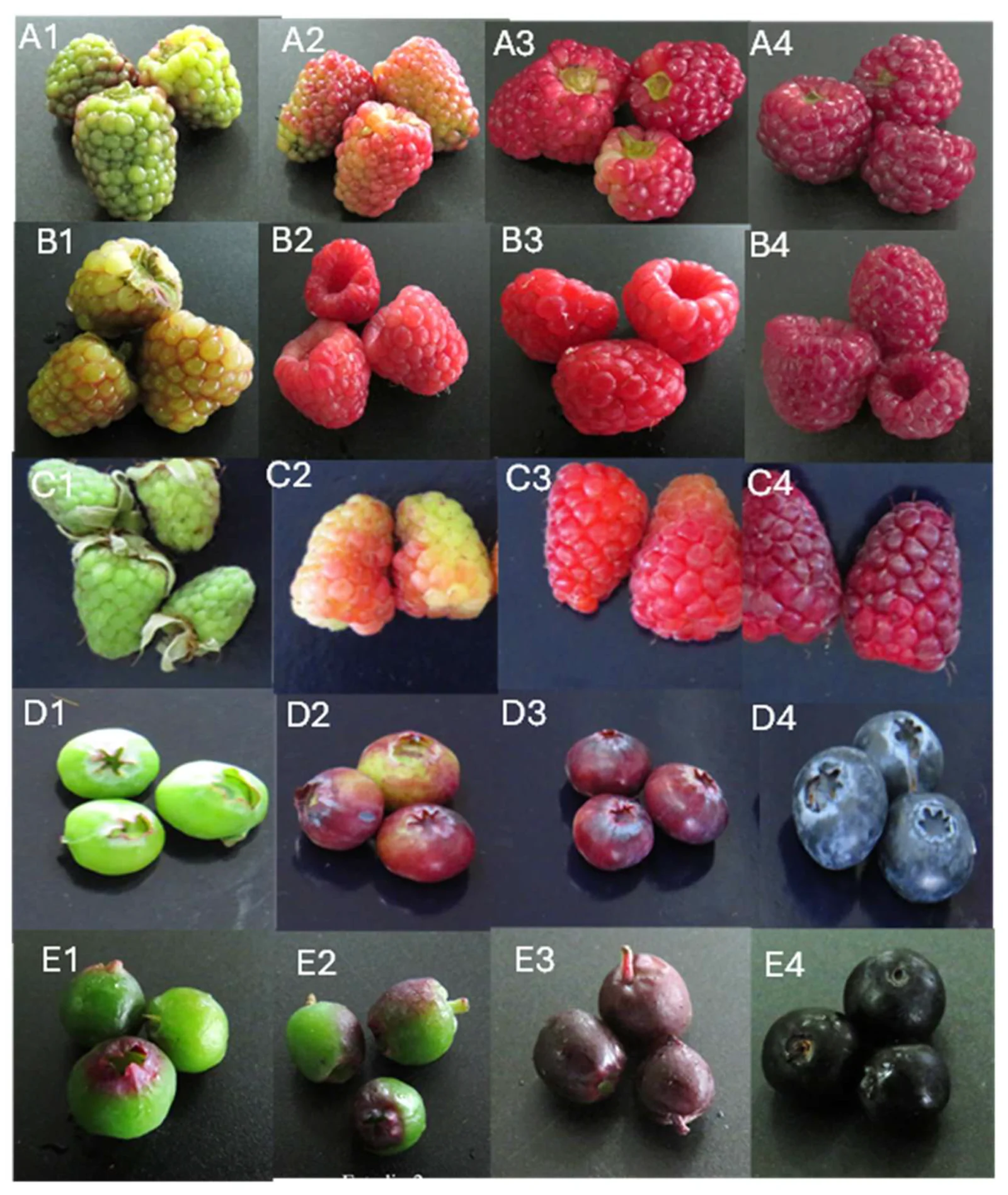
Red berries at different degrees of ripeness. Note: (**A1**–**A4**) Blackberry ‘Castilla’ (*Rubus glaucus*) (code 4508); (**B1**–**B4**) Raspberry ‘Buckingham Tayberry’ (*Rubus × hybrid*) (code 4504); (**C1**–**C4**) Raspberry ‘Heritage’ (*Rubus idaeus*) (code 4509); (**D1**–**D4**) Blueberry (*Vaccinium corymbosum*) (code 4506); (**E1**–**E4**) ‘Mortiño’ (*Vaccinium meridionale*) (code 4507). (**A1**), M0% Blackberry; (**A2**), M50% Blackberry; (**A3**), M80% Blackberry; (**A4**), M100% Blackberry; (**B1**), M0% Raspberry; (**B2**), M50% Raspberry; (**B3**), M80% Raspberry; (**B4**), M100% Raspberry; (**C1**), M0% Raspberry; (**C2**), M50% Raspberry; (**C3**), M80% Raspberry; (**C4**), M100% Raspberry; (**D1**), M0% Blueberry; (**D2**), M50% Blueberry; (**D3**), M80% Blueberry; (**D4**), M100% Blueberry; (**E1**), M0% ‘Mortiño’; (**E2**), M50% ‘Mortiño’; (**E3**), M80% ‘Mortiño’; (**E4**), M100% ‘Mortiño’.

This study considered five varieties of red fruit at different stages of ripeness (Figure 1). Botanical specimens’ representative of each type of fruit was collected and identified by the Herbarium of the Salesian Polytechnic University in Quito, Ecuador, on the basis of morphological descriptors and/or taxonomic keys. The reference specimens were deposited under the following accession numbers: Blackberry ‘Castilla’ (*Rubus glaucus*) (code 4508); Raspberry ‘Buckingham Tayberry’ (*Rubus × hybrid*) (code 4504); Raspberry ‘Heritage’ (*Rubus idaeus*) (code 4509); Blueberry (*Vaccinium corymbosum*) (code 4506); and ‘Mortiño’ (*Vaccinium meridionale*) (code 4507). The collection and analysis of the plant material were conducted under the framework contract MAE-DNB-CMD-2017-0050-UTE and Research Project MAE-DNB-2019-0911-O. These authorisations ensured compliance with the applicable Ecuadorian regulations governing access to and research on biological diversity.

The samples were collected from plantations in the province of Cotopaxi in Ecuador. To minimise the variability associated with crop management and growing conditions, one orchard was selected for each fruit type. Within each orchard, 40 trees located in the central rows were selected in order to reduce any potential edge effects. Three composite biological samples, prepared separately, were collected for each fruit type and stage of ripeness. Plant samples were collected for botanical identification, and sampling was random, with approximately 2 kg of fruit collected per variety. One composite sample corresponded to each stage of ripening. The biological samples were standardised by composite mass rather than by a fixed number of fruits. Each ripening stage was harvested on a different date between January and June 2022 and was never combined across stages.

Ripeness stages were established using the number of days after pollination (DAP). For ‘Castilla’ blackberry (*Rubus glaucus*), samples were assigned to M0%, M50%, M80%, and M100% using the objective colour categories established in the Ecuadorian standard NTE-INEN-2427 [20]. For ‘Buckingham Tayberry’ raspberry (*Rubus* × *hybrid*), the stages were established as M0% at 20 DAP, M50% at 36–45 DAP, M80% at 46–55 DAP, and M100% at 56–60 DAP. For ‘Heritage’ raspberry (*Rubus idaeus*), M0% corresponded to 15 DAP, M50% to 26–35 DAP, M80% to 36–45 DAP, and M100% to 46–50 DAP. For blueberry (*Vaccinium corymbosum*), M0% corresponded to 20 DAP, M50% to 41–60 DAP, M80% to 61–80 DAP, and M100% to 81–90 DAP. For ‘Mortiño’ (*Vaccinium meridionale*), M0% corresponded to 30–40 DAP, M50% to 45–65 DAP, M80% to 70–80 DAP, and M100% to 85–90 DAP. Samples from each stage were collected on separate dates according to these developmental periods.

The sample collected was divided into two portions. The first, consisting of fresh fruit, was used to assess commercial quality by determining weight, size (equatorial and longitudinal diameters), pH, total soluble solids, titratable acidity, moisture, and ash content. The second portion was frozen at −80 °C and then freeze-dried in a Christ Alpha 1–4 LDplus (Martin Christ Gefriertrocknungsanlagen GmbH, Osterode am Harz, Germany). The dried samples were finely ground, the seeds were separated, and the resulting powders were stored in hermetically sealed amber bottles until analysis.

#### Mineral Profile

The quantification of calcium, iron, potassium, magnesium, and sodium in the freeze-dried samples was performed using microwave-assisted acid digestion, followed by atomic absorption spectrophotometry. For digestion, 40 mg of dry sample was weighed into digestion vessels of the Speed-180 Wave Xpert Microwave system (Berghof Products + Instruments GmbH, Eningen unter Achalm, Germany). A total of 5 mL of nitric acid (65%) was added to each sample and left to stand for 10 min to allow for initial decomposition of the organic material. Subsequently, a staged digestion programme was applied, which included 140 °C, 30 bar, and 70% power for 5 min; 200 °C, 35 bar, and 80% power for 15 min; and 50 °C, 25 bar, without power for 10 min to complete the decomposition. Once the process was complete, the containers were cooled to room temperature for 20 min, and the resulting solutions were transferred to 25 mL volumetric flasks, with the volume made up with ultrapure water Milli-Q (Merck KGaA, Darmstadt, Germany) [21].

The mineral content was determined using a Varian SpectrAA-55 atomic absorption spectrophotometer (Agilent Technologies, Mississauga, ON, Canada), using differential wavelengths for each mineral (Ca (422.7 nm), Fe (372.0 nm), Mg (202.6 nm), K (766.5 nm) and Na (589.6 nm)). A mixture of acetylene and nitrous oxide was used for calcium analysis, while a mixture of acetylene and air was used for the other elements.

The calibration curves were prepared from standard solutions at 1000 ppm, with concentration ranges of 0–5 ppm for Ca, 0–20 ppm for Fe, 0–200 ppm for K, 0–10 ppm for Mg, and 0–8 ppm for Na. Each determination was performed in triplicate, and the results were expressed as milligrams of mineral per 100 g of dry weight (mg/100 g DW).

### 2.2. Analysis of Bioactive Compounds

#### 2.2.1. Vitamin C

Vitamin C quantification was performed using 40 mg of lyophilised powder, which was mixed with 1.2 mL of 3% metaphosphoric acid and 0.2 mL of a 0.2% *DL*-homocysteine solution. The mixture was homogenised and subjected to ultrasonic agitation in an FS60 bath (Fisher Scientific Inc., Waltham, MA, USA) for one minute to promote extraction. The volume was then made up to 2 mL with the same extraction solvent [21].

The samples were filtered through a 0.45 µm PVDF filter and placed in vials for chromatographic analysis. The determination was carried out on an RRLC 1200 rapid resolution liquid chromatography system (Agilent Technologies, Mississauga, ON, Canada) equipped with a DAD-UV/Vis detector set at 244 nm and a Zorbax Eclipse C18 column (1.8 µm, 4.6 mm × 50 mm) (Agilent Scientific Instruments, Santa Clara, CA, USA).

The mobile phase consisted of a mixture of 1.5% monobasic potassium phosphate and 1.8% *n*-acetyl-*n*,*n*,*n*-trimethylammonium bromide, dissolved in methanol (90:10, *v*/*v*), with a constant flow rate of 1 mL/min. The calibration curve was prepared using a standard *L*-ascorbic acid solution at 0.1 mg/mL. To plot the calibration curve, between 1 and 20 µL of the standard solution was injected. The calibration curve had an R^2^ of 0.99. The method’s limits of detection (LODs) and quantification (LOQs) were 0.20 ppm and 0.65 ppm, respectively. Each sample was extracted in triplicate, and each extract was injected in duplicate. The results were expressed as milligrams of vitamin C per 100 g of dry weight (mg/100 g DW).

#### 2.2.2. Organic Acid Profile

Organic acid quantification was performed using 20 mg of lyophilised powder, which was mixed with 1.5 mL of 0.02 N sulphuric acid, in the presence of 0.05% metaphosphoric acid and 0.02% *DL*-homocysteine. The mixture was homogenised and subjected to ultrasonic agitation for 3 min to promote extraction. The volume was then made up to 2 mL with deionised water [21].

The mixture was filtered through a 0.45 µm PVDF filter and placed in vials for chromatographic analysis. The determination was carried out on an RRLC 1200, equipped with a DAD-UV/Vis detector set at 210 nm and a YMC-Triart C18 column (3 µm, 4.6 mm × 150 mm) (YMC Europe GmbH, Dinslaken, Germany). The mobile phase consisted of a 0.027% sulphuric acid with a constant flow rate of 1 mL/min. The calibration curve was prepared using standard solutions of malic, citric, and tartaric acids at 0.1 mg/mL. To plot the calibration curve, between 1 and 20 µL of the standard solution was injected separately. The calibration curve had an R^2^ of 0.99. LODs and LOQs were 0.06 ppm and 0.17 ppm for tartaric acid, 0.08 and 0.26 ppm for citric acid, and 0.13 and 0.39 ppm for malic acid, respectively. Each sample was extracted in triplicate, and each extract was injected in duplicate. The results were expressed as milligrams of organic acid per 100 g of dry weight (mg/100 g DW).

#### 2.2.3. Carotenoid, Chlorophyll, and Their Derivative Profiles

Carotenoid profile quantification was performed using 20 mg of lyophilised powder, which was mixed with methanol, dichloromethane, and acetone in a 1:2:1 ratio. The mixture was homogenised and subjected to ultrasonic agitation for 2 min to promote extraction. The coloured phase was recovered by centrifugation in an Eppendorf 5430 (Eppendorf AG, Hamburg, Germany) at 14,000 rpm for 5 min at 4 °C, and the solid residue was subjected to successive extractions with the same solvents until complete recovery of the pigments was ensured. The coloured extract was evaporated to dryness in a Büchi R-100 rotary evaporator (Fisher Scientific, Hampton, NH, USA) at a temperature not exceeding 40 °C. The dry extract was redissolved with 40 µL of ethyl acetate. The solids were removed by centrifugation (14,000 rpm, 7 min, 4 °C), and 20 µL of the liquid was placed in a vial insert for chromatographic analysis [22].

Determination was carried out on an RRLC 1200, equipped with a DAD-UV/Vis detector set between 250 and 500 nm and a YMC C30 column (3 µm, 4.6 × 150 mm) (YMC Europe GmbH, Dinslaken, Germany). The mobile phase consisted of a linear gradient of acetonitrile (A), methanol (B) and ethyl acetate (C) with a constant flow rate of 1 mL/min of 0–5 min, 85% of A + 15% of B; 5–7 min, 60% A + 20% B + 20%C; 7–12 min, 85% A + 15% B. The calibration curve was prepared using standard solutions of astaxanthin, β-carotene, β-cryptoxanthin, lutein, lycopene, zeaxanthin, trans-β-apo-8′-carotenal, and α-carotene at 0.1 mg/mL. To plot the calibration curve, between 1 and 20 µL of the standard solution was injected separately. The calibration curve had an R^2^ of 0.99. LODs and LOQs were 0.007 and 0.02 ppm for lutein, 0.029 and 0.09 ppm for β-carotene, and 0.003 and 0.008 ppm for zeaxanthin, respectively. Each sample was extracted in triplicate, and each extract was injected in duplicate. The results were expressed as milligrams of carotenoid per 100 g of dry weight (mg/100 g DW). The same method was used to determine and quantify chlorophylls and their derivatives. Standards of chlorophyll a and b and pheophytin a and b were used for the analysis.

#### 2.2.4. Phenolic Profile

Phenolic quantification was performed using 20 mg of lyophilised powder, which was mixed with 1 mL of methanol acidified with 0.1% HCl. The mixture was homogenised and subjected to ultrasonic agitation for 3 min to promote extraction. The organic phase was recovered by centrifugation (14,000 rpm, 5 min, 4 °C), and the solid residue was extracted twice more with 500 mL of the methanolic solution. The mixture was filtered through a 0.45 µm PVDF filter and placed in vials for chromatographic analysis [22].

Determination was carried out on an RRLC 1200, equipped with a DAD-UV/Vis detector set between 200 and 500 nm and a Zorbax Eclipse Plus C18 column (4.6 × 150 mm, 5 µm) (Agilent Scientific Instruments, Santa Clara, CA, USA). The mobile phase consisted of a linear gradient of 0.01% aqueous formic acid (solvent A) and acetonitrile (solvent B) with a constant flow rate of 1 mL/min from 0 min, 100% A; 5 min, 95% A + 5% B; 20 min, 50% A + 50% B; 30 min. The calibration curve was prepared using a standard solution of caffeic acid, chlorogenic acid, chrysin, *p*-, *m*- and *o*-coumaric acid, ferulic acid, gallic acid, *p*-hydroxybenzoic acid, 3-hydroxybenzoic acid, 2-methoxybenzoic acid, 3-methoxybenzoic acid, 2,5-dihydroxybenzoic acid, kaempferol, luteolin, naringin, quercetin, rutin, shikimic acid, syringic acid and vanillic acid at 0.1 mg/mL. To plot the calibration curve, between 1 and 20 µL of the standard solution was injected separately. The calibration curve had an R^2^ of 0.99. LODs and LOQs were 0.009 ppm and 0.028 ppm for chlorogenic acid, 0.048 ppm and 0.145 ppm for caffeic acid, 0.007 ppm and 0.021 ppm for gallic acid, respectively. Each sample was extracted in triplicate, and each extract was injected in duplicate. The results were expressed as milligrams of phenolics per 100 g of dry weight (mg/100 g DW).

#### 2.2.5. Total Anthocyanins

Anthocyanin quantification was performed using 40 mg of lyophilised powder, which was mixed with 2 mL of 96% ethanol. The mixture was homogenised and subjected to ultrasonic agitation for 3 min to promote extraction. The mixture was filtered through a 0.45 µm PVDF filter. To determine the total anthocyanin content, two 50 µL aliquots of the supernatant were taken and placed in separate wells of a 96-well plate. In the first well, 200 µL of a potassium chloride buffer solution (0.025 M, pH 1.0) was added, while in the second well, 200 µL of a sodium acetate buffer solution (0.4 M, pH 4.5) was added.

The absorbances at 520 nm and 700 nm were recorded using a spectrophotometer with a microplate reader. Quantification was performed using a standard curve of cyanidin-3-glucoside over the concentration range of 0.05 to 0.20 mg/mL. Each sample was extracted in triplicate, and each extract was read twice. The results were expressed as cyanidin-3-glucoside equivalents per 100 g dry weight (mg C3G/100 g DW) [22].

### 2.3. Antioxidant Activity Analyses

Antioxidant activity was quantified using 20 mg of lyophilised powder, mixed with 2 mL of methanol. The mixture was homogenised and subjected to ultrasonic agitation for 3 min to promote extraction. The mixture was filtered through a 0.45 µm PVDF filter [23].

Antioxidant capacity was evaluated using methods based on the ABTS•^+^ and DPPH• radicals. The ABTS•^+^ radical was generated by mixing equal volumes of 2.45 mM potassium persulfate and 7 mM ABTS, then incubating the mixture for 16 h in the dark at room temperature. The resulting solution was diluted with HPLC-grade methanol until the absorbance at 734 nm reached 0.70 ± 0.02. For analysis, 10 µL of the extract or standard was mixed with 200 µL of the ABTS•^+^ radical solution in a 96-well plate. Methanol, radical, and extract blanks were included. Absorbance was measured at 734 nm using a microplate reader BioTek (Agilent Scientific Instruments, Santa Clara, CA, USA). The calibration curve was constructed using a 10 mM Trolox standard, with concentrations ranging from 0.2 to 0.7 mM.

For the DPPH• radical assay, a solution was prepared by dissolving 10 mg of DPPH in 50 mL of HPLC-grade methanol. A total of 20 µL of the extract or standard and 280 µL of the DPPH• solution were added to each well of the microplate. The blanks correspond to methanol, radical, and extract. The plates were incubated in the dark for 30 min, and readings were taken at 515 nm using the same equipment. The calibration curve was prepared using a 10 mM Trolox standard, diluted to 0.4–4.0 mM.

The results of both methods were expressed as millimoles of Trolox equivalent per 100 g of dry weight (mmol TE/100 g DW).

### 2.4. Antimicrobial Activity Analyses

Antimicrobial activity was evaluated at M0% and M100% as an endpoint screening strategy. These stages were selected because they represent contrasting physiological and chemical profiles: early stages may contain higher concentrations of several non-anthocyanin phenolics, whereas advanced ripening generally favours anthocyanin accumulation [14]. Given the large panel of fruit–microorganism combinations, the two extreme stages were initially screened to identify extracts with sufficiently low MIC values to justify subsequent stage-resolved analyses. Therefore, the antimicrobial assays were not designed to determine the complete trajectory of antimicrobial activity throughout ripening.

#### 2.4.1. Antibacterial Activity

The following bacterial strians were used to evaluate antimicrobial activity through the determination of the Minimum Inhibitory Concentration (MIC): *Escherichia coli* ATCC 8739, *Staphylococcus aureus* ATCC 6538P, *Pseudomonas aeruginosa* ATCC 9027, *Streptococcus mutans* ATCC 25175, *Enterococcus faecalis* ATCC 49533, *Listeria monocytogenes* ATCC 19114, *Klebsiella pneumoniae* ATCC 10031, *Cutibacterium acnes* ATCC 11827, *Porphyromonas gingivalis* ATCC 33277, *Staphylococcus epidermidis* ATCC 12228, *Pseudomonas fluorescens* ATCC 13525, *Pseudomonas putida* ATCC 31483, and *Edwardsiella tarda* ATCC 15947. The bacterial panel was selected to include both clinically relevant pathogens and environmental or food-associated microorganisms.

The extract was prepared by homogenising 2.5 g of lyophilised plant material in 25 mL of 50% ethanol. To enhance compound recovery, the mixture was sonicated for 6 min in an ultrasonic bath (Model F560, Scientific, Waltham, MA, USA). Samples were then centrifuged at 7500 rpm for 5 min at 4 °C (Eppendorf, Bochum, Germany). The supernatant was carefully separated, and the extraction process was repeated twice with fresh solvent to ensure exhaustive recovery of soluble metabolites. The combined filtrates were passed through 0.45 µm PVDF syringe filters and concentrated under reduced pressure using a rotary evaporator, maintaining temperatures below 40 °C to prevent compound degradation. The concentrated extract was frozen and subsequently lyophilised using a Christ Alpha 1–4 LDplus freeze dryer (Martin Christ Gefriertrocknungsanlagen GmbH, Bochum, Germany). The resulting powder was stored frozen until analysis. The stock extract solutions were prepared at a target concentration of approximately 300 mg/mL in sterile distilled water. Owing to small variations in the mass of lyophilised extract weighed, the actual stock concentrations ranged approximately from 300 to 305 mg/mL. For the antimicrobial assay, 100 µL of the stock extract solution was combined with 100 µL of the standardised microbial inoculum, resulting in a final volume of 200 µL per well and an initial extract concentration of approximately 150 mg/mL. Twofold serial dilutions subsequently yielded nominal final concentrations of 150, 75, 37.5, 18.75, 9.375, 4.6875, 2.34375, and 1.171875 mg/mL. For extracts whose stock concentration differed slightly from 300 mg/mL, the corresponding final concentrations were calculated individually from the actual concentration of the prepared stock. The lyophilised extract was completely reconstituted in sterile distilled water at a concentration of 300 mg/mL, producing a visually homogeneous solution without visible precipitate or phase separation before preparation of the serial dilutions. Therefore, the use of DMSO or another organic cosolvent was not required. The initial concentration was selected based on solubility and to cover a range compatible with the expected activity levels of crude botanical extracts, typically within the mg/mL scale.

Antibacterial activity was determined using the broth microdilution method in accordance with the recommendations of the Clinical and Laboratory Standards Institute (CLSI) [24]. For *E. coli*, *S. aureus*, *P. aeruginosa*, *S. mutans*, *S. epidermidis*, *P. fluorescens*, *P. putida*, *E. faecalis*, *K. pneumoniae*, *L. monocytogenes*, *P. gingivalis*, *and C. acnes* and *E. tarda*, cultures were grown overnight in Brain Heart Infusion (BHI) broth at 37 °C with constant agitation or 48 h at 37 °C with 5% CO_2_ (*P. gingivalis* and *C. acnes*). Serial dilutions of the extract were prepared directly in 96-well microplates by mixing 100 µL of the extract solution with 100 µL of inoculum BHI medium giving an antimicrobial incubation volume of 200 µL per well. The recorded final inoculum was approximately 5 × 10^5^ CFU/mL per well The final extract series during incubation was 150, 75, 37.5, 18.75, 9.375, 4.6875, 2.34375, and 1.171875 mg/mL. For samples whose initial stock concentration differed slightly from 150 mg/mL, the actual concentrations were calculated individually from the experimentally recorded stock concentration. Plates were incubated for 24 h at 37 °C. Streptomycin (1.56 mg/mL) was used as the positive control, while sterile water served as the negative control.

For growth evaluation via colorimetric reading, the TTC reagent (2,3,5-triphenyltetrazolium chloride) was added to the microplate at the end of the incubation period. 20 μL was added, and the plate was incubated at 37 °C in the dark for 1 to 2 h. TTC acts as a redox indicator that, when reduced by metabolically active viable cells, transforms into a visible red colouration, which allows for visual determination of growth. The post-incubation TTC addition was not used to calculate the antimicrobial concentrations reported above.

Aliquots of 5 μL from each well were subcultured onto Brain Heart Infusion agar plates previously gridded at their base. Each sample was deposited in an identified section of the quadrant. Plates were incubated under the same conditions as the initial cultures to assess the extracts’ ability to inhibit bacterial growth. This recovery check was not interpreted as an MBC because quantitative viable-count reduction criteria were not measured.

The MIC was defined as the lowest extract concentration at which no bacterial growth was observed under the assay conditions. All tests and assays were performed in duplicate to ensure reliability and reproducibility.

#### 2.4.2. Antifungal Activity

Representative fungal strains were included to assess antifungal activity, including *Candida albicans* ATCC 1031, *Candida tropicalis* ATCC 13803, *Aspergillus niger* ATCC 6275, *Fusarium keratoplasticum* ATCC 36031, *Malassezia furfur* ATCC 14521, *Trichophyton interdigitale* ATCC 9533, and *Microsporum canis* ATCC 36299.

For inoculum preparation, fungal cultures were propagated in yeast peptone dextrose broth (YPDB) to obtain actively growing cells. Extract preparation and the broth microdilution procedure were performed as described for the antibacterial assay (Section 2.4.1).

Serial dilutions of the extract were prepared in 96-well microplates by mixing 100 µL of extract solution with 100 µL of inoculated BHI medium. Twofold dilution yielded final extract concentrations of 150, 75, 37.5, 18.75, 9.375, 4.6875, 2.34375, and 1.171875 mg/mL. The final inoculum was adjusted to approximately 0.5–2.5 × 10^3^ CFU/mL per well for yeasts and 0.4–5 × 10^4^ CFU/mL per well for filamentous fungi A separate viable-count verification of the final CFU/mL was not performed in the original screening. Plates were incubated for 24 h at 37 °C. Fluconazole (1.5 mg/mL) was used as the positive control, while sterile water served as the negative control.

Growth was evaluated using the TTC reagent (2,3,5-triphenyltetrazolium chloride), which was incorporated into the microplate at the end of the incubation period, and the plate was incubated at 37 °C in the dark for 1 to 2 h. TTC acts as a redox indicator that, when reduced by metabolically active viable cells, turns red, allowing for visual determination of growth. The MIC was defined as the lowest extract concentration required to inhibit growth completely. All assays were performed in triplicate to ensure reliability and reproducibility.

For filamentous fungi and dermatophytes (*Trichophyton interdigitale*, *Aspergillus niger*, *Fusarium keratoplasticum*, *Microsporum cani* and *Malassezia furfur*), assays were performed by combining equal volumes of extract solution and fungal inoculum in YPDB medium directly within the wells of a 96-well microplate to perform serial twofold dilutions. The final inoculum density was approximately 1 × 10^6^ CFU/mL. *F. keratoplasticu*, *T. interdigitale*, *M. canis* and *A. niger* were incubated at 25 °C for 5 days, and *M. furfur* was incubated at 30 °C for 5 days.

Fluconazole served as the reference antifungal drug (positive control), whereas the negative control consisted of inoculated wells containing sterile water instead of extract. Subsequently, after 24 h of incubation, 4 μL of the contents of each well is taken and inoculated into a Petri dish with Sabouraud dextrose agar (SDA) previously gridded at its base. Each aliquot was deposited in an identified section of the quadrant. The box is incubated at 37 °C for 24 h to confirm inhibition of yeast growth by the evaluated extracts. The grid arrangement facilitates visual comparison, revealing differences in the density, colouration, and morphology of the colonies, thereby enabling a qualitative evaluation complementary to the quantitative data. The MIC was defined as the lowest extract concentration that completely inhibited fungal growth, and all experiments were performed in triplicate.

### 2.5. Statistical Analysis

Results are expressed as the mean ± standard deviation of the independent biological replicates. Each parameter was analysed separately. Statistical significance between maturity stages was evaluated by one-way analysis of variance (simple ANOVA). The mean separation was made via Tukey’s test with 0.05 significant differences. In the tables, different lowercase letters indicate significant differences among ripeness stages within the same fruit. The row labelled “Among-fruit comparison at M0%” reports the significance of the comparison among fruit types at the M0% ripeness stage. Statistical significance is denoted as follows: ns, not significant; con, *p* > 0.05; *, *p* < 0.05; **, *p* < 0.01; and ***, *p* < 0.001. Each parameter was analysed independently across four maturity levels (M0%, M50%, M80%, and M100%) for Blackberry ‘Castilla’, Raspberry ‘Buckingham Tayberry’, Raspberry ‘Heritage’, Blueberry and ‘Mortiño’.

Principal component analysis (PCA) was performed to explore the multivariate structure of the data and to identify patterns of variation among samples based on their physicochemical characteristics. Prior to the analysis, all variables were standardised by centring at the mean and scaling to unit variance, to eliminate the influence of different measurement units and ensure equal contribution of each variable to the model. The PCA enabled visualisation of relationships between fruit types and maturity stages, highlighting the principal dimensions that explain the dataset’s variance. This approach facilitated the identification of the variables that contributed most to sample differentiation.

A hierarchical clustering heatmap was constructed to visualise standardised biochemical profiles and biological activities across fruit types and maturity stages. Raw data were averaged per fruit–maturity group, and variables with zero variance were excluded. The remaining features were scaled using z-score normalisation and clustered using Euclidean distance with complete-linkage. Sample annotations were colour-coded by fruit type and maturity level. Quantitative associations among bioactive compounds, antioxidant capacity, and antimicrobial responses were evaluated using Spearman rank correlations. Data analysis was carried out using RStudio 4.4.1 for principal component analysis, correlations and heat mapping, and Statgraphics Centurion XVII and SigmaPlot 14.0 for ANOVA analysis and mean separation; these programmes were used for organising and visualising the data, as well as for inferential statistics.

## 3. Results and Discussion

### 3.1. Physicochemical Characteristics

Table 1 presents the physicochemical characteristics and mineral composition of the five fruits at four stages of ripening.

Ripening affected fruit mass more consistently than fruit dimensions. ‘Castilla’ blackberry and ‘Buckingham Tayberry’ raspberry showed progressive increases in weight and reached their highest values at M100%, whereas ‘Heritage’ raspberry and blueberry reached their maximum weight at M80%. ‘Mortiño’ remained the smallest fruit but showed a marked increase in weight at M100%. Changes in longitudinal and equatorial diameters were less uniform, indicating that increases in fruit mass and external dimensions did not occur proportionally in all fruits.

Soluble solids showed the clearest shared response to ripening. Despite intermediate fluctuations, all fruits presented higher values at M100% than at M0%. The increase was progressive in ‘Castilla’ blackberry and ‘Buckingham Tayberry’ raspberry, whereas ‘Heritage’ raspberry and blueberry reached their highest concentrations at M80%, followed by a slight reduction at M100%. ‘Mortiño’ differed by showing little variation between M0% and M80%, followed by a pronounced increase at full ripeness. These patterns demonstrate that soluble-solid accumulation occurred at different rates among the fruits.

In contrast, titratable acidity did not decrease uniformly. ‘Heritage’ raspberry, blueberry, and ‘Mortiño’ showed lower acidity at M100% than at M0%, although the changes across intermediate stages differed. In ‘Castilla’ blackberry, acidity increased at M50% and M80% before declining at M100%, whereas ‘Buckingham Tayberry’ raspberry showed a progressive increase during ripening. The pH also varied within relatively narrow ranges and did not consistently follow the changes in titratable acidity. Therefore, although increasing soluble solids and decreasing acidity are commonly associated with berry ripening, the present results demonstrate that this relationship was fruit-dependent. The increase in soluble solids observed in ‘Castilla’ blackberry agrees with previous results for *Rubus glaucus*; however, the intermediate increase in acidity found in the present study indicates that the temporal pattern was not identical [10].

Moisture and ash content also showed fruit-specific responses. Moisture increased towards advanced ripening stages in ‘Castilla’ blackberry and ‘Mortiño’, whereas the other fruits exhibited fluctuations without a common directional trend. Ash content generally decreased from M0% to M100% in ‘Castilla’ blackberry, ‘Buckingham Tayberry’ raspberry, and ‘Mortiño’, while changes in ‘Heritage’ raspberry and blueberry were comparatively smaller. These results indicate that ripening affected the overall proportion of inorganic material differently among fruits.

Mineral composition displayed greater variability than the conventional ripening indicators. Calcium decreased markedly during ripening in ‘Castilla’ blackberry and ‘Buckingham Tayberry’ raspberry and remained lower at the advanced stages in ‘Heritage’ raspberry. Conversely, blueberry showed higher calcium concentrations from M50% onwards, while ‘Mortiño’ reached its highest value at M50% before decreasing at M100%. Magnesium generally declined with ripening, particularly in the two raspberries and blueberry. Potassium also tended to be lower at advanced maturity, although intermediate increases or partial recoveries occurred in some fruits. Iron and sodium followed irregular patterns, with no common response across species.

The mineral profiles reported by other authors for *Vaccinium* and *Rubus* fruits also vary considerably among studies [25,26]. Furthermore, a literature review also highlights the variability in the concentration of different minerals in red fruits [1,2,5]. These comparisons are for reference purposes only, as mineral concentrations depend on the fruit species, cultivar, stage of ripeness and production conditions. Under these common analytical conditions, the results demonstrate that the effect of ripening was specific to each mineral and each fruit, rather than following a single pattern of accumulation or depletion.

### 3.2. Analysis of Bioactive Compounds and Antioxidant Activity

Table 2 presents the concentrations of vitamin C and the principal organic acids, together with the antioxidant capacity determined using the DPPH• and ABTS•^+^ assays. Significant differences were observed among fruit types and ripeness stages. Nevertheless, ripening did not produce a common response because the direction and magnitude of the changes depended on the fruit, compound, and antioxidant assay.

Vitamin C followed contrasting trajectories. In ‘Castilla’ blackberry, its concentration decreased between M0% and M80% and subsequently increased markedly at M100%. ‘Buckingham Tayberry’ raspberry reached its highest concentration at M50%, followed by a reduction at M80% and a partial recovery at M100%. In contrast, ‘Heritage’ raspberry showed a progressive increase up to M80% and maintained a comparatively high concentration at M100%. Vitamin C was not detected in blueberry from M0% to M80% and was only detected at full ripeness. ‘Mortiño’ contained low concentrations during the early stages, followed by an increase at M80% and M100%.

Comparison among fruits showed that ‘Heritage’ raspberry had the highest vitamin C concentration at every ripeness stage, reaching 56.9 mg/100 g DW at M80%. At M100%, it was followed by ‘Castilla’ blackberry and ‘Buckingham Tayberry’, whereas blueberry and ‘Mortiño’ showed considerably lower concentrations. Therefore, advanced ripening favoured vitamin C accumulation in ‘Castilla’ blackberry, blueberry, and ‘Mortiño’, while both raspberry cultivars reached their maximum concentrations before full ripeness.

Previous studies have reported vitamin C concentrations of 34–54 mg/100 g FW in blackberries, 5–92.2 mg/100 g FW in raspberries, and 10–100 mg/100 g FW in blueberries. Differences among studies may also be associated with botanical material, ripeness stage, geographical origin, cultivation conditions, and analytical methodology [1]. Other reviews have also documented considerable variability in the vitamin and bioactive composition of red fruits [5]. Differences among studies may also be associated with botanical material, ripeness stage, geographical origin, cultivation conditions, and analytical methodology [1]. In Andean blackberry, altitude and developmental stage have been associated with differences in chemical composition and antioxidant capacity [14].

Organic acid composition also differed among fruits. Citric acid predominated in ‘Castilla’ blackberry and both raspberries, whereas malic acid made a greater contribution to the organic acid profile of ‘Mortiño’. In blueberry, malic acid was predominant at M0%, but both citric and malic acids decreased markedly towards full ripeness. These results are consistent with the literature identifying citric and malic acids as the principal organic acids in berries, although their relative proportions vary among species [1].

The response of individual organic acids provided more information than their total concentration. In ‘Castilla’ blackberry, citric acid generally increased during ripening, whereas malic and tartaric acids decreased towards M100%; consequently, total organic acids were higher at full ripeness than at M0%. ‘Buckingham Tayberry’ and ‘Heritage’ raspberries reached their highest total organic acid concentrations at M50%, followed by decreases towards M100%. In both fruits, this reduction was principally associated with declining malic and tartaric acid concentrations. Blueberry showed the clearest decrease in total organic acids, driven mainly by the pronounced loss of malic acid and the reduction in citric acid at M100%. Conversely, ‘Mortiño’ showed an increase in malic acid at M80% and M100%, while tartaric acid progressively decreased. Therefore, the commonly described reduction in organic acids during ripening was evident in blueberry and at the advanced stages of both raspberries but was not applicable to ‘Castilla’ blackberry or ‘Mortiño’.

Previous studies have reported approximately 0.8 g/100 g of organic acids in blueberries and 1.9 g/100 g in raspberries [2]. Citric acid concentrations of 13.4–75.11 mg/g DW and malic acid concentrations of 1.0–7.21 mg/100 g DW have also been described in blueberries [1]. These broad ranges show that comparisons depend strongly on the analytical and expression basis. Moreover, the citric and malic acid concentrations reported in the literature differ substantially among blueberry materials, supporting the species-, cultivar-, and environment-dependent behaviour observed in the present study.

The ABTS•^+^ assay also revealed fruit-specific responses. ‘Castilla’ blackberry showed a progressive reduction from M0% to M100%, while ‘Heritage’ raspberry presented an overall decrease with intermediate fluctuations. In contrast, ‘Buckingham Tayberry’ raspberry showed a non-linear response, with its lowest value at M80% and its highest at M100%. Blueberry remained comparatively stable across the four stages, whereas ‘Mortiño’ showed a slight increase at full ripeness. The different trajectories obtained using DPPH• and ABTS•^+^ are expected because the assays differ in their reaction conditions and sensitivity to compounds with different physicochemical properties. Nevertheless, the present data only demonstrate differences between the two chemical assays and do not identify which individual compounds were responsible for those responses.

For ‘Castilla’ blackberry, the reduction in ABTS•^+^ capacity towards full ripeness agrees directionally with previous results for *Rubus glaucus*, in which antioxidant capacity decreased from 701.6 µmol TE/g DW (70.2 mmol TE/100 g DW) at 25% ripeness to 658.3 µmol TE/g DW (65.8 mmol TE/100 g DW) at full ripeness [10]. Although the direction of change was similar, the difference in magnitude may be related to plant material, cultivation environment, extraction procedure, and analytical conditions. Accordingly, the results should be considered directionally consistent but not quantitatively equivalent.

Antioxidant capacity determined by DPPH• followed different trajectories among fruits. ‘Buckingham Tayberry’ raspberry showed a progressive increase from 4.9 mmol TE/100 g DW at M0% to 6.1 mmol TE/100 g DW at M100%. Blueberry also showed an overall increase, although with an intermediate reduction at M80%, and reached its highest value at full ripeness. In contrast, ‘Castilla’ blackberry decreased from M0% to M80% and showed only slight recovery at M100%. ‘Heritage’ raspberry followed a similar pattern, with a progressive reduction until M80% followed by a partial recovery in ripe fruit. ‘Mortiño’ showed the highest initial DPPH• value, decreased until M80%, and increased again at M100%. Therefore, ripening enhanced DPPH• antioxidant capacity in ‘Buckingham Tayberry’ and, overall, in blueberry, whereas the remaining fruits showed reductions followed by partial recovery or stabilisation.

The comparison among fruits also changed with ripeness. At M0%, ‘Mortiño’ presented the highest DPPH• value and ‘Buckingham Tayberry’ the lowest. At M50%, blueberry showed the highest numerical value, while ‘Heritage’ raspberry showed the lowest. At M80%, no significant differences were detected among the five fruits. At M100%, ‘Buckingham Tayberry’ and blueberry presented the highest values, followed closely by ‘Mortiño’, whereas ‘Castilla’ blackberry and ‘Heritage’ raspberry showed lower antioxidant capacity. These results confirm that the effect of ripening on DPPH• activity was fruit-dependent rather than uniform across red fruits.

Overall, ripening produced compound- and fruit-dependent responses rather than a common bioactive or antioxidant pattern. Vitamin C increased at advanced stages in several fruits, whereas organic acids decreased markedly in blueberry and in both raspberries after M50% but followed different trajectories in ‘Castilla’ blackberry and ‘Mortiño’. Antioxidant capacity also varied according to fruit, ripening stage, and assay and did not consistently follow vitamin C or total organic acid concentrations.

#### 3.2.1. Carotenoid, Chlorophyll, and Derivative Profiles

Table 3 presents the carotenoid, chlorophyll, and chlorophyll-derivative profiles of the five fruits throughout ripening. The carotenoids identified included α-carotene, β-carotene, lutein, 9-*cis*-antheraxanthin, zeaxanthin, and zeinoxanthin. However, their occurrence and trajectories differed considerably among fruits, demonstrating that ripening did not produce a common carotenoid accumulation pattern.

‘Castilla’ blackberry showed a progressive reduction in total carotenoids from M0% to M100%, primarily reflecting the decrease in lutein, which was the only carotenoid detected in this fruit. ‘Mortiño’ showed a similar overall reduction, although its initial profile was more diverse and included lutein, 9-cis-antheraxanthin, and zeaxanthin. In ‘Buckingham Tayberry’ raspberry, total carotenoids also decreased overall, but the trajectory was not strictly progressive because a partial increase occurred at M80%, coinciding with the detection of 9-cis-antheraxanthin, zeaxanthin, and zeinoxanthin.

In contrast, ‘Heritage’ raspberry accumulated carotenoids as ripening progressed. The increase observed from M50% onwards was mainly associated with β-carotene, while α-carotene was detected only at M80% and M100%. Consequently, ‘Heritage’ raspberry showed the highest total carotenoid concentrations among the fruits evaluated, particularly at M50% and M100%. This pattern differed markedly from the overall reductions observed in ‘Castilla’ blackberry, ‘Buckingham Tayberry’ raspberry, and ‘Mortiño’.

Blueberry showed a biphasic response. Total carotenoids initially decreased at M50% but subsequently increased at M80% and M100%. This late accumulation was principally associated with lutein and 9-*cis*-antheraxanthin. Therefore, the blueberry response differed from the progressive carotenoid loss observed in ‘Castilla’ blackberry and ‘Mortiño’, but it also differed compositionally from ‘Heritage’ raspberry, in which β-carotene was the predominant carotenoid at advanced ripening stages.

Chlorophylls and their derivatives also followed fruit-specific trajectories. Their total concentration decreased markedly during ripening in ‘Castilla’ blackberry, ‘Buckingham Tayberry’ raspberry, and ‘Mortiño’. In these fruits, the reduction was primarily associated with the decline in pheophytin b and, when detected, chlorophyll b. In ‘Heritage’ raspberry, chlorophyll-related compounds were detected only at the early stages, supporting the loss of green pigments during ripening.

The blueberry profile was notably different. After an initial reduction at M50%, the total concentration of chlorophylls and derivatives increased at M80% and reached its highest value at M100%. The reduction in chlorophyll-related compounds observed in ‘Castilla’ blackberry, ‘Buckingham Tayberry’ raspberry, and ‘Mortiño’ is consistent with the general pigment changes associated with berry ripening. In Andean blackberry, early developmental stages are characterised by green pigments, whereas advanced ripening is accompanied by changes in pigment composition and increasing anthocyanin accumulation [14]. Previous reviews have also shown that carotenoid concentrations and profiles differ substantially among red fruits and are influenced by botanical identity, cultivar, ripening stage, geographical origin, cultivation conditions, and analytical methodology [1,5].

Overall, ripening promoted three contrasting pigment patterns, such as a progressive decline in carotenoids and chlorophyll-related compounds in ‘Castilla’ blackberry and ‘Mortiño’; an overall decline with intermediate fluctuations in ‘Buckingham Tayberry’ raspberry; and carotenoid accumulation at advanced ripening stages in ‘Heritage’ raspberry and blueberry. These findings reinforce the fruit- and compound-dependent nature of pigment transformations during ripening.

#### 3.2.2. Phenolic Profile and Total Anthocyanins

Table 4 shows the phenolic concentration profile and total anthocyanin content of the major phenolic compounds identified in samples analysed at different ripeness stages. The main compounds detected were gallic acid, 4-hydroxybenzoic acid, m-coumaric acid, syringic acid, chlorogenic acid, caffeic acid, ferulic acid, kaempferol, quercetin glucoside, and quercetin.

In ‘Castilla’ blackberry, total phenolics fluctuated during development and reached their highest concentration at M100% (709.9 mg/100 g DW). This increase was mainly associated with the marked accumulation of *m*-coumaric acid in ripe fruit, whereas caffeic and ferulic acids decreased. Total anthocyanins increased from 23.7 to 231.2 mg/100 g DW between M0% and M100%, consistent with the progressive accumulation of these pigments previously reported during the ripening of *Rubus* fruits [8,14].

Both raspberry cultivars showed increasing total phenolic concentrations during ripening, although their individual profiles differed. In ‘Buckingham Tayberry’, total phenolics increased from 130.7 mg/100 g DW at M0% to 646.5 mg/100 g DW at M100%, primarily reflecting the substantial accumulation of chlorogenic acid in the final stage. Total anthocyanins also increased, reaching 113.6 mg/100 g DW at M100%. In ‘Heritage’, total phenolics increased from 91.7 to 392.8 mg/100 g DW, accompanied by a progressive increase in *m*-coumaric acid. Its anthocyanin concentration was also highest in ripe fruit (112.4 mg/100 g DW). Thus, despite belonging to the same genus, the two raspberry cultivars differed in the phenolic compounds that contributed most to the increase observed during ripening.

Blueberry displayed the opposite trend for non-anthocyanin phenolics. Total phenolics decreased from 3453.0 mg/100 g DW at M0% to 934.3 mg/100 g DW at M100%, together with reductions in chlorogenic, caffeic, and ferulic acids, kaempferol, and both forms of quercetin. Nevertheless, total anthocyanins increased markedly and reached 512.9 mg/100 g DW at M100%. Therefore, the decline in the quantified phenolic acids and flavonols did not prevent anthocyanin accumulation during blueberry ripening.

‘Mortiño’ contained the highest total phenolic concentrations among the fruits analysed, reaching a maximum of 5005.2 mg/100 g DW at M50%. Caffeic and ferulic acids were the principal contributors at the early and intermediate stages. Total phenolics subsequently decreased to 2018.4 mg/100 g DW at M100%, whereas anthocyanins increased sharply to 970.7 mg/100 g DW, the highest concentration recorded among the ripe fruits. This contrasting behaviour demonstrates that the evolution of total phenolics cannot be inferred solely from anthocyanin accumulation.

The compounds identified agree qualitatively with the phenolic classes reported for *Rubus* and *Vaccinium* fruits, which include hydroxybenzoic and hydroxycinnamic acids, flavonols, and anthocyanins [2,5]. Overall, ripening favoured anthocyanin accumulation in all the fruits studied, whereas the remaining phenolic compounds followed fruit-specific trajectories: total phenolics increased in both raspberry cultivars, decreased in blueberry, peaked at an intermediate stage in ‘Mortiño’, and fluctuated in ‘Castilla’ blackberry.

The relationship between phenolic composition and antioxidant capacity was not uniform across fruits. In ‘Castilla’ blackberry, total phenolics and anthocyanins increased at M100%, whereas ABTS•^+^ decreased and DPPH• remained below its M0% value. This apparent discrepancy reflects a change in phenolic composition: the increase in total phenolics was mainly driven by *m*-coumaric acid, while caffeic acid, ferulic acid, kaempferol, and quercetin glucoside decreased during ripening. A similar divergence was observed in ‘Heritage’ raspberry, where total phenolics and anthocyanins increased, but antioxidant capacity remained lower at M100% than at M0%. Conversely, ‘Buckingham Tayberry’ showed concurrent increases in phenolics, anthocyanins, and antioxidant capacity at full ripeness. Blueberry and ‘Mortiño’ presented intermediate patterns: anthocyanins increased markedly, but non-anthocyanin phenolics decreased or peaked before full ripeness, while antioxidant capacity remained stable or fluctuated depending on the assay.

These findings indicate that antioxidant capacity depends on phenolic composition rather than exclusively on the total concentration. Individual phenolic compounds differ in their radical scavenging reactivity; consequently, anthocyanin accumulation may not fully compensate for reductions in other phenolic acids or flavonols. In Andean blackberry, non-anthocyanin polyphenols predominate at early developmental stages and contribute substantially to antioxidant capacity, whereas anthocyanins become predominant during ripening [14].

Therefore, an increase in anthocyanins does not necessarily produce a proportional increase in the overall antioxidant response. The divergence between DPPH• and ABTS•^+^ also reflects the assay-dependent nature of antioxidant measurements. Compound structure, steric accessibility, reaction kinetics, and solvent conditions affect the response to DPPH•, while ABTS•^+^ can react with a broader range of hydrophilic and lipophilic antioxidants. Thus, changes in the relative proportions of vitamin C, phenolic acids, flavonols, anthocyanins, and carotenoids may affect each assay differently, even when the summed concentration of bioactive compounds increases [27,28]. These chemical assays measure the combined response of the extract and cannot identify the contribution of each compound independently.

### 3.3. Antimicrobial Activity Analyses

Table 5 shows the minimum inhibitory concentration (MIC) of the dry ethanolic extracts of the samples under study, evaluated at 0% and 100% maturity, which are considered the extremes of the fruit’s physiological behaviour but do not establish a continuous maturity–activity relationship. These stages were selected as an initial endpoint screening because they represent contrasting physiological and bioactive profiles: early stages may contain higher concentrations of several non-anthocyanin phenolics, whereas advanced ripening generally favours anthocyanin accumulation [14]. The tests were performed against Gram-positive and Gram-negative bacteria, as well as pathogenic yeasts. MIC values ranged from 1.2 to 151.5 mg/mL, with lower values indicating greater antimicrobial activity.

For most bacterial strains, the extracts from ‘Heritage’ raspberry, blueberry, and ‘Mortiño’ showed lower activity at M100% than at M0%, as indicated by increased MICs. This reduction was particularly evident against *Edwardsiella tarda*, *Pseudomonas aeruginosa*, *Staphylococcus aureus*, and *S. epidermidis*. A similar tendency was observed in ‘Castilla’ blackberry and ‘Buckingham Tayberry’ raspberry, although several microorganism-specific exceptions occurred.

Ripening enhanced antibacterial activity in some combinations. The MIC of ‘Castilla’ blackberry decreased from 2.3 to 1.2 mg/mL against *Cutibacterium acnes* and from 37.5 to 18.8 mg/mL against *Klebsiella pneumoniae*. ‘Buckingham Tayberry’ showed a pronounced improvement against *Porphyromonas gingivalis*, for which the MIC decreased from 112.5 to 7.0 mg/mL. The ripe blueberry and ‘Mortiño’ extracts were more active against *Escherichia coli*, with MICs decreasing from 37.5 to 18.8 mg/mL in both fruits. These exceptions demonstrate that the influence of ripening was microorganism-dependent and cannot be described solely as a general loss of antibacterial activity.

Among the unripe fruits, particularly low MICs were recorded for ‘Buckingham Tayberry’ against *K. pneumoniae* (2.3 mg/mL), ‘Castilla’ blackberry against *Listeria monocytogenes* and *P. gingivalis* (3.5 mg/mL), and ‘Mortiño’ against *Streptococcus mutans* (1.2 mg/mL). At M100%, the lowest antibacterial MIC was observed for ‘Castilla’ blackberry against *C. acnes* (1.2 mg/mL). Thus, the fruit extract showing the greatest activity differed according to the target bacterium.

Antifungal activity also varied among species and ripeness stages. None of the extracts inhibited *Aspergillus niger* at the concentrations tested. Activity against *Candida albicans* and *C. tropicalis* was limited or undetectable, particularly for both raspberry cultivars. In contrast, all extracts were active against *Malassezia furfur*, with MICs of 1.2 mg/mL, except ripe ‘Buckingham Tayberry’, which showed an MIC of 2.3 mg/mL. The extracts also inhibited the dermatophytes *Microsporum canis* and *Trichophyton interdigitale*, although ripening generally reduced activity against *M. canis*, except in ‘Castilla’ blackberry and ‘Buckingham Tayberry’.

Anthocyanin-rich fractions from Ecuadorian *Rubus glaucus* and *Vaccinium floribundum* have previously shown an MIC of 100 mg/mL against *C. albicans* ATCC 10231 [29]. In the present study, ripe ‘Castilla’ blackberry and ‘Mortiño’ extracts showed MICs of 37.5 and 75.0 mg/mL, respectively. Nevertheless, these values should be compared cautiously because the studies differed in the type of extract or fraction and, consequently, in chemical composition.

The antimicrobial results did not show a consistent correspondence with total phenolic or anthocyanin concentrations. Although ‘Mortiño’ contained the highest total phenolic and anthocyanin concentrations, it did not consistently present the lowest MICs. Likewise, phenolics and anthocyanins increased at M100% in ‘Castilla’ blackberry, but its antimicrobial activity improved against some microorganisms and decreased against others. Moreover, associations between phenolic composition and antimicrobial activity have been reported for berry extracts [30,31].

The differences observed between species and ripeness stages may be due to several factors, including the fruit matrix (skin, seed, pulp), which can influence the concentration and accessibility of antimicrobial compounds. The specific variety and genotype determine the phenol profile and functional capacity. In addition, crop altitude and agronomic conditions (light, temperature, soil) influence the biosynthesis of phenolic compounds responsible for antimicrobial activity [32]. The degree of ripeness influences maturation, as degradation, conjugation, or transformation of phenols and anthocyanins can occur, thereby decreasing antimicrobial activity even if the fruit appears more ripe or visually appealing [33,34].

Although no visible precipitation was observed during extract reconstitution, macroscopic homogeneity does not confirm the molecular solubilisation of every minor lipophilic constituent. Therefore, the MIC values represent the antimicrobial activity of the compounds that remained dissolved or uniformly dispersed under the aqueous assay conditions. Furthermore, the omission of M50% and M80% is a limitation because some compounds reached their maximum concentrations at intermediate stages. In particular, total phenolics in ‘Mortiño’ peaked at M50%, and extracts from this stage could present antimicrobial responses that cannot be predicted from the M0% and M100% results. Future studies should therefore evaluate all four ripeness stages, prioritising the fruit–microorganism combinations with the lowest endpoint MICs, and examine their relationships with individual bioactive compounds using independent biological replicates.

### 3.4. Statistical Analysis

**Figure 2:**
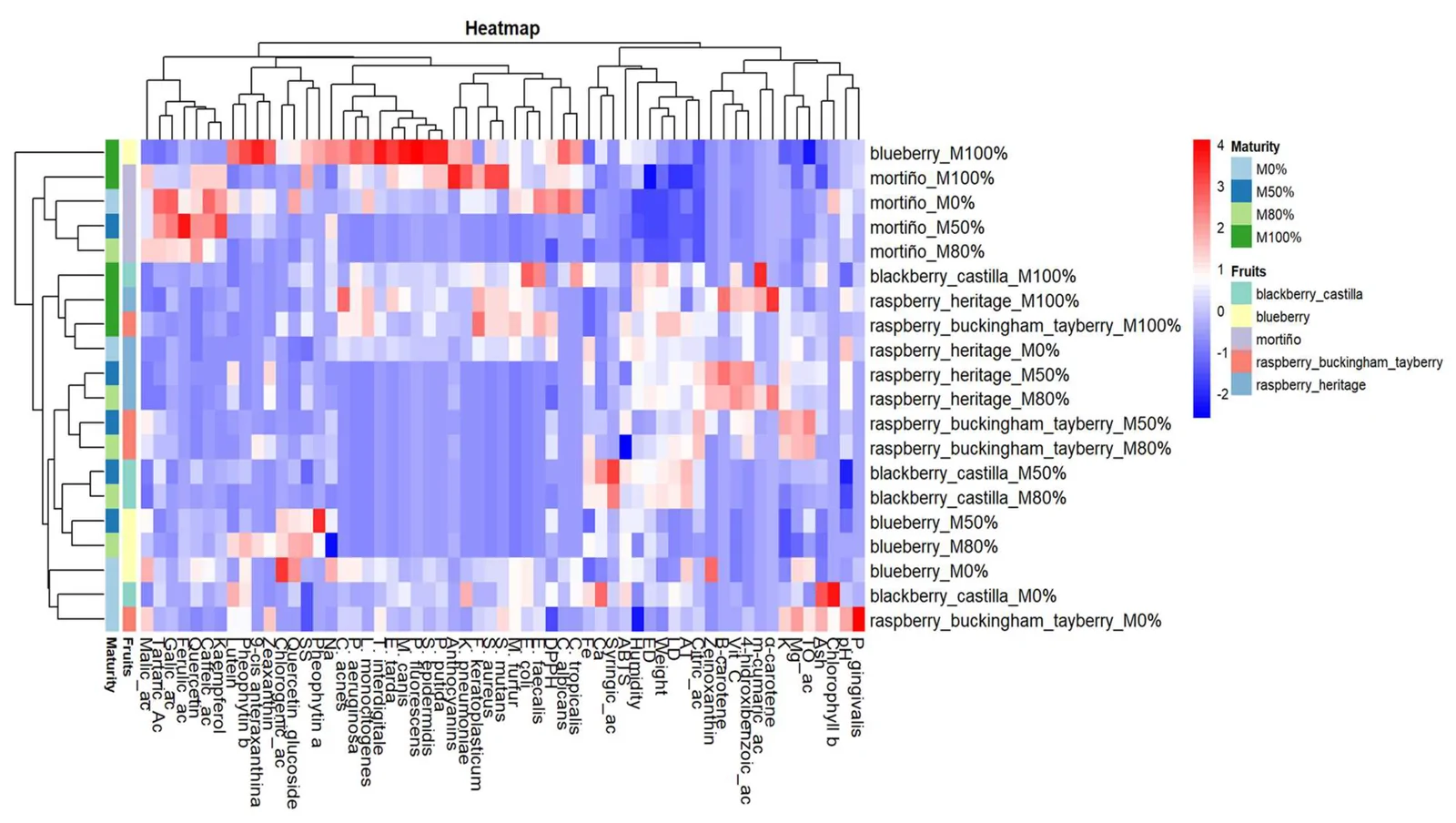
A dendrogram of red berries based on the variables analysed at different degrees of ripeness. **Note:** Chlorogenic_ac (chlorogenic acid), caffeic_ac (caffeic acid), ferulic_ac (ferulic acid), galic_ac (gallic acid), citric_ac (citric acid), malic_ac (malic acid), tartaric_ac (tartaric acid), syringic_ac (syringic acid), 4-hidroxibenzoic_ac (4-hydroxybenzoic acid), *m*-cumaric_ac (*m*-coumaric acid), and TO_ac (*o*-coumaric acid), LD (longitudinal diameter), ED (equatorial diameter), SS (soluble solids), AT (titratable acidity), vitamin C (Vit_C), calcium (Ca), iron (Fe), potassium (K), magnesium (Mg), and sodium (Na), *Escherichia coli* (*E. coli*), *Staphylococcus aureus* (*S. aureus*), *Pseudomonas aeruginosa* (*P. aeruginosa*), *Streptococcus mutans* (*S. mutans*), *Enterococcus faecalis* (*E. faecalis*), *Listeria monocytogenes* (*L. monocytogenes*), *Klebsiella pneumoniae* (*K. pneumoniae*), *Cutibacterium acnes* (*C. acnes*), *Porphyromonas gingivalis* (*P. gingivalis*), *Staphylococcus epidermidis* (*S. epidermidis*), *Pseudomonas fluorescens* (*P. fluorescens*), *Pseudomonas putida* (*P. putida*), *Edwardsiella tarda* (*E. tarda*), *Candida albicans* (*C. albicans*), *Candida tropicalis* (*C. tropicalis*), *Aspergillus niger* (*A. niger*), *Fusarium keratoplasticum* (*F. keratoplasticum*), *Malassezia furfur* (*M. furfur*), *Microsporuma canis* (*M. canis*), and *Trichophyton interdigitale* (*T. interdigitale*).

Figure 2 shows the heatmap generated from the standardised physicochemical, compositional, antioxidant, and antimicrobial variables. The hierarchical clustering differentiated the samples according to both fruit type and ripeness stage. Samples at M80% and M100% generally showed greater proximity, whereas several M0% and M50% samples formed separate clusters, indicating that ripening was an important—but not exclusive—source of variation. Samples with a maturity level of M0% to M50% formed separate clusters, reflecting a metabolism dominated by early structural and defensive compounds, whereas during ripening, biology reprogrammes metabolism towards sugars, pigments, and volatiles that attract dispersers [35].

‘Mortiño’ showed the clearest separation among ripeness stages, consistent with its marked changes in phenolic composition and anthocyanin concentration. However, the phenolic variables did not follow a common trajectory in all fruits. Caffeic and chlorogenic acids decreased during blueberry ripening, whereas *m*-coumaric acid increased in ripe ‘Castilla’ blackberry and ‘Heritage’ raspberry. Therefore, the clustering reflects fruit-specific compositional changes rather than a generalised decrease in phenolic acids. Differences among species remained evident within similar ripeness stages, particularly because ‘Mortiño’ and blueberry contained higher concentrations of several phenolic compounds than the *Rubus* fruits.

Thus, ‘Mortiño’ shows a more pronounced transition between maturity states, suggesting more intense metabolic dynamics, particularly in phenolic compounds and anthocyanins, as reported by other authors [29]. On the other hand, although maturity dominates the grouping, subclusters are observed by species. For example, ‘Mortiño’ has a higher overall polyphenol content; blueberries have a more homogeneous profile, with a predominance of anthocyanins; and raspberries and blackberries have a greater diversity of flavanol and phenolic acid compounds.

**Figure 3:**
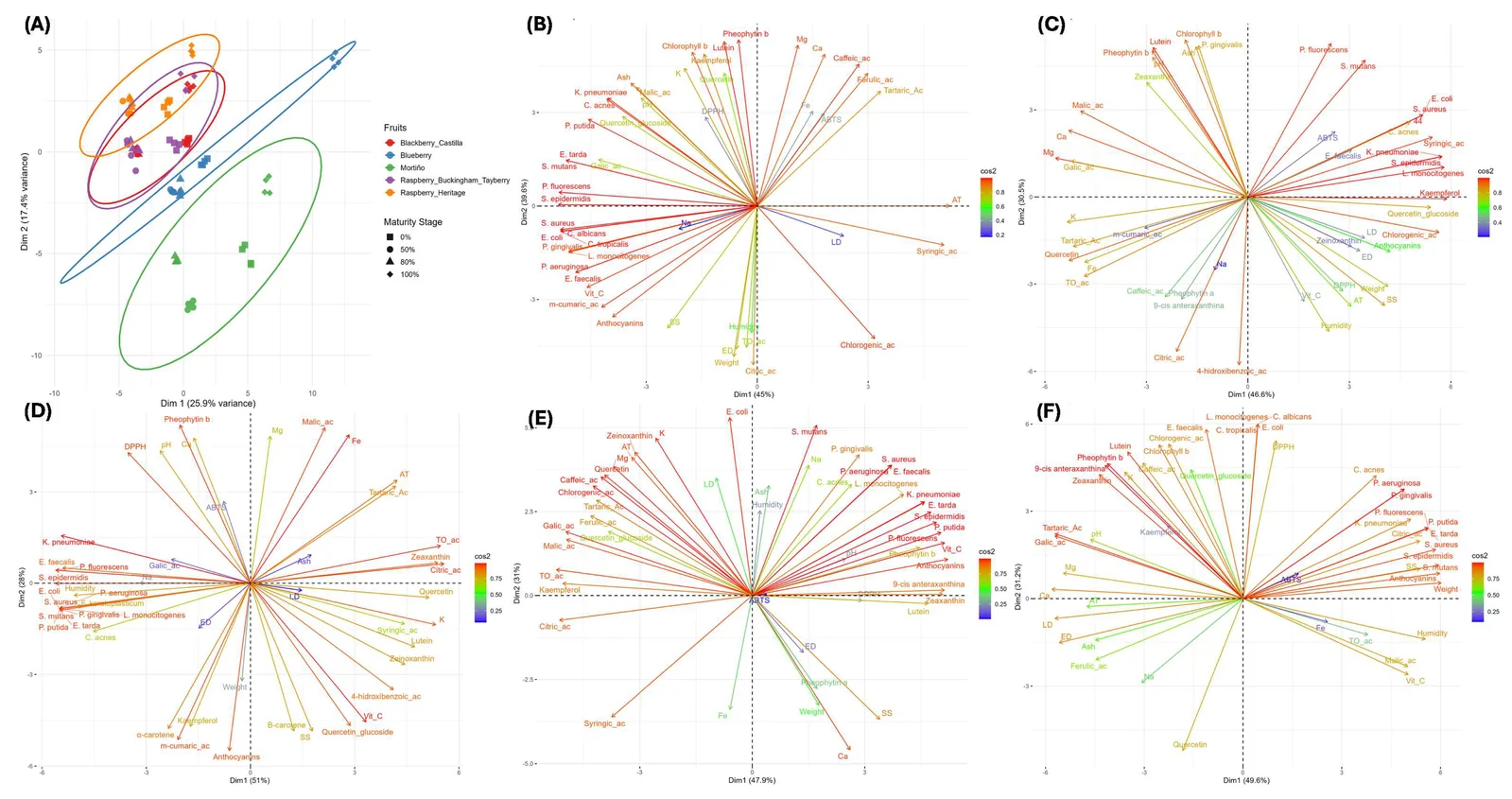
PCA of red berries at different degrees of ripeness. (**A**) PCA grouped by fruit type and degree of ripeness; (**B**) Blackberry ‘Castilla’ (*Rubus glaucus*); (**C**) Raspberry ‘Buckingham Tayberry’ (*Rubus × hybrid*); (**D**) Raspberry ‘Heritage’ (*Rubus idaeus*); (**E**) Blueberry (*Vaccinium corymbosum*); (**F**) ‘Mortiño’ (*Vaccinium meridionale*). Note: Chlorogenic_ac (chlorogenic acid), caffeic_ac (caffeic acid), ferulic_ac (ferulic acid), galic_ac (gallic acid), citric_ac (citric acid), malic_ac (malic acid), tartaric_ac (tartaric acid), syringic_ac (syringic acid), 4-hidroxibenzoic_ac (4-hydroxybenzoic acid), *m*-cumaric_ac (*m*-coumaric acid), and TO_ac (*o*-coumaric acid), LD (longitudinal diameter), ED (equatorial diameter), SS (soluble solids), AT (titratable acidity), vitamin C (Vit_C), calcium (Ca), iron (Fe), potassium (K), magnesium (Mg), and sodium (Na), *Escherichia coli* (*E. coli*), *Staphylococcus aureus* (*S. aureus*), *Pseudomonas aeruginosa* (*P. aeruginosa*), *Streptococcus mutans* (*S. mutans*), *Enterococcus faecalis* (*E. faecalis*), *Listeria monocytogenes* (*L. monocytogenes*), *Klebsiella pneumoniae* (*K. pneumoniae*), *Cutibacterium acnes* (*C. acnes*), *Porphyromonas gingivalis* (*P. gingivalis*), *Staphylococcus epidermidis* (*S. epidermidis*), *Pseudomonas fluorescens* (*P. fluorescens*), *Pseudomonas putida* (*P. putida*), *Edwardsiella tarda* (*E. tarda*), *Candida albicans* (*C. albicans*), *Candida tropicalis* (*C. tropicalis*), *Aspergillus niger* (*A. niger*), *Fusarium keratoplasticum* (*F. keratoplasticum*), *Malassezia furfur* (*M. furfur*), *Microsporum canis* (*M. canis*), and *Trichophyton interdigitale* (*T. interdigitale*).

The overall PCA is presented in Figure 3A. DIM 1 and DIM 2 explained 25.9% and 17.4% of the total variance, respectively, accounting jointly for 43.3%. This percentage was sufficient for an exploratory two-dimensional visualisation of the main patterns, but it did not capture most of the dataset’s variability because 56.7% was retained in subsequent components. ‘Mortiño’ was separated from most of the other samples and showed broad dispersion among ripeness stages. Blueberry samples were primarily distributed along the positive side of DIM 1, whereas ‘Castilla’ blackberry occupied a more central position. The two raspberry cultivars showed partial overlap, indicating similarities in the variables included in the analysis. Their proximity may be consistent with their taxonomic relationship within *Rubus* [36], but the PCA alone does not demonstrate that genetic relatedness caused the observed grouping.

The species-specific PCAs explained a higher proportion of variance in their first two dimensions (Figure 3B–F): 84.6% for ‘Castilla’ blackberry (DIM 1: 45.0%; DIM 2: 39.6%), 77.1% for ‘Buckingham Tayberry’ (46.6%; 30.5%), 79.0% for ‘Heritage’ raspberry (51.0%; 28.0%), 80.4% for blueberry (47.9%; 31.0%), and 81.4% for ‘Mortiño’ (49.0%; 32.4%). These values indicate that the two-dimensional biplots retained most of the variability within each fruit. Nevertheless, each species-specific PCA was based on only four ripeness stages; consequently, these projections should be considered exploratory. The proximity of phenolic variables to the antioxidant assays is consistent with the antioxidant properties reported for phenolic compounds [37,38].

These distributions support the differentiation between variables associated with advanced ripening and those occurring at higher levels during earlier stages. Several antimicrobial variables were positioned near physicochemical or bioactive compound vectors in the species-specific biplots. However, this proximity indicates covariation and does not demonstrate that any individual compound caused the antimicrobial response. Natural flavonoids have shown antibacterial properties; specifically, quercetin enhanced the activity of polymyxin E against MCR-1-positive *Escherichia coli*, rather than promoting bacterial growth. Antimicrobial effects have also been reported for organic acids [39,40,41].

**Figure 4:**
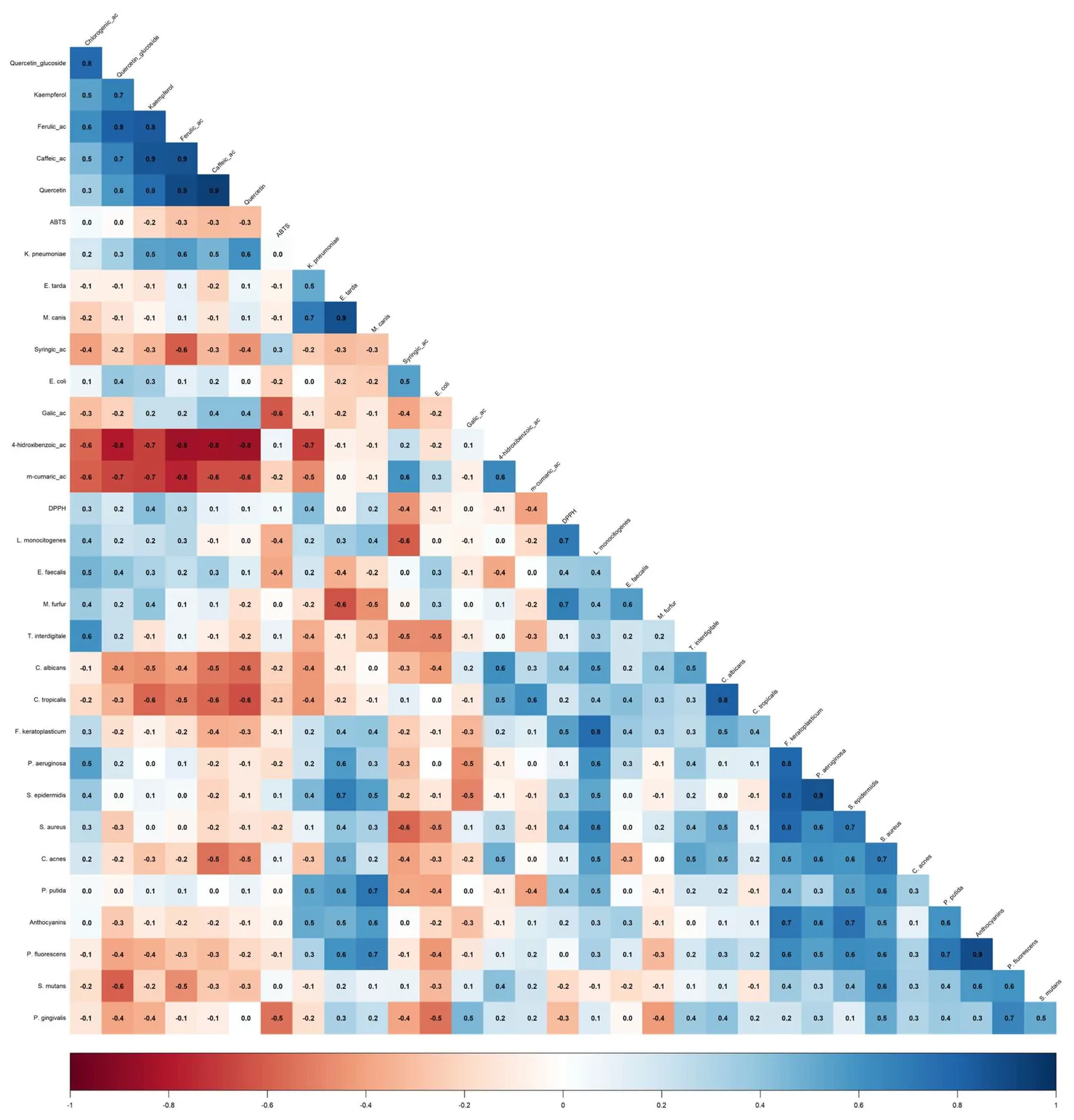
Spearman rank correlation matrix of Andean red fruit extracts. **Note:** Chlorogenic_ac (chlorogenic acid), Caffeic_ac (caffeic acid), Ferulic_ac (ferulic acid), m-coumaric_ac (*m*-coumaric acid), 4-HBA (4-hydroxybenzoic acid), Kaempferol (kaempferol), Quercetin (quercetin), Q_glucoside (quercetin glucoside), Anthocyanin (total anthocyanin content), DPPH (2,2-diphenyl-1-picrylhydrazyl assay), ABTS (2,2′-azino-bis(3-ethylbenzothiazoline-6-sulfonic acid) assay), E_coli (*Escherichia coli*), S_aureus (*Staphylococcus aureus*), P_aeruginosa (*Pseudomonas aeruginosa*), E_faecalis (*Enterococcus faecalis*), L_monocytogenes (*Listeria monocytogenes*), K_pneumoniae (*Klebsiella pneumoniae*), C_albicans (*Candida albicans*), C_tropicalis (*Candida tropicalis*), M_furfur (*Malassezia furfur*), F_solani (*Fusarium keratoplasticum*), T_mentagrophytes (*Trichophyton interdigitale*), and P_gingivalis (*Porphyromonas gingivalis*).

Spearman correlation analysis showed clear covariation among several phenolic compounds (Figure 4). Caffeic acid was strongly correlated with ferulic acid, quercetin, and kaempferol (ρ = 0.9), while ferulic acid was also associated with quercetin (ρ = 0.9), kaempferol (ρ = 0.8), and quercetin glucoside (ρ = 0.8). These relationships support the coordinated phytochemical patterns previously observed in the heatmap and PCA. In contrast, antioxidant capacity showed a less uniform association with phenolic composition. DPPH and ABTS were weakly correlated (ρ = 0.1); ABTS showed an inverse relationship with gallic acid (ρ = −0.6), whereas DPPH was positively associated with kaempferol (ρ = 0.4). Anthocyanins showed weak relationships with DPPH (ρ = 0.2) and ABTS (ρ = 0.0), indicating that antioxidant capacity was not determined by a single phenolic class.

Relationships with antimicrobial susceptibility were also compound- and microorganism-dependent. Negative correlations indicate associations with lower MICs and therefore greater inhibitory activity. Relevant inverse relationships included 4-hydroxybenzoic acid with *K. pneumoniae* (ρ = −0.7), syringic acid with *S. aureus* and *L. monocytogenes* (ρ = −0.6), and quercetin with *C. albicans* and *C. tropicalis* (ρ = −0.6). Caffeic acid was also inversely associated with *C. tropicalis* (ρ = −0.6) and *C. albicans* (ρ = −0.5). These patterns are consistent with the selective antimicrobial responses observed among the extracts, although the strong covariation among phenolic compounds prevents attribution of the effect to individual metabolites.

Anthocyanins showed positive correlations with several MIC endpoints, particularly *P. fluorescens* (ρ = 0.9), *F. keratoplasticum* and *S. epidermidis* (ρ = 0.7), and *P. aeruginosa*, *P. putida*, and *S. mutans* (ρ = 0.6). Thus, higher anthocyanin levels were not necessarily associated with stronger antimicrobial activity. Together with their weak relationships with DPPH and ABTS, this finding reinforces the multivariate results by showing that ripening-related changes in phytochemical composition do not produce a uniform change in biological activity. Rather, antioxidant and antimicrobial responses appear to depend on the overall chemical profile and on the specific biological endpoint evaluated.

Taken together, the results indicate that fruit identity exerted a stronger influence than a common ripening pattern on the physicochemical, phytochemical, antioxidant, and antimicrobial responses. This interpretation is also supported by the predominantly species-level clustering observed in the heatmap. The differences among fruits may reflect their distinct phytochemical profiles and species-specific ripening metabolism; however, the present design does not allow the respective contributions of genotype, cultivation conditions, and geographical origin to be separated. Consequently, the selection of a harvest stage should be based on the specific fruit and intended compositional or biological characteristic rather than on the assumption that full ripeness is universally optimal.

### 3.5. Study Limitations and Future Perspectives

This study has several limitations. Antimicrobial activity was evaluated using crude hydroethanolic extracts; therefore, the observed MIC values cannot be attributed to individual compounds, and possible synergistic or antagonistic interactions among extract constituents remain unresolved. Moreover, antimicrobial assays were conducted only at M0% and M100%, preventing identification of potential activity maxima at the intermediate ripeness stages. The assays were exclusively *in vitro*, and cytotoxicity, selectivity indices, stability, sensory effects, and efficacy in food matrices were not evaluated. Consequently, the results do not demonstrate clinical efficacy, activity against antimicrobial-resistant infections, or direct suitability as food preservatives. Similarly, DPPH and ABTS are cell-free chemical assays and do not establish antioxidant effects in biological systems. The global PCA retained 43.3% of the variance in its first two dimensions, while the species-specific analyses included only four ripeness stages; therefore, these multivariate patterns should be considered exploratory and not causal. Future studies should include bioassay-guided fractionation, identification of active constituents, evaluation of intermediate ripeness stages, cytotoxicity and selectivity testing, and validation in cellular systems and food matrices.

## 4. Conclusions

Red fruits are widely recognised for their health-promoting properties, and numerous studies have examined changes in their phytochemical composition and biological activities during ripening. The present study complements this evidence by comparing five fruits at four ripeness stages using the same analytical approach. Ripening produced fruit-dependent changes in the physicochemical characteristics, bioactive composition, antioxidant capacity, and antimicrobial activity of the five fruits evaluated. Soluble solids were generally higher at advanced ripeness stages, whereas titratable acidity decreased in most fruits, except ‘Buckingham Tayberry’. The behaviour of vitamin C, organic acids, carotenoids, and chlorophyll-related compounds also differed among fruits, demonstrating that full ripeness did not maximise all compositional characteristics. At M100%, total phenolic concentrations were higher than at M0% in ‘Castilla’ blackberry and both raspberry cultivars, but lower in blueberry and ‘Mortiño’. In contrast, anthocyanin concentrations were higher in ripe fruits in all cases, with ‘Mortiño’ showing the greatest accumulation. ‘Mortiño’ also presented the highest total phenolic concentration, although its maximum occurred at M50% rather than at full ripeness. Antioxidant capacity did not consistently parallel vitamin C, total phenolics, or anthocyanins and depended on both fruit type and analytical assay. Antimicrobial activity was likewise specific to the fruit, ripeness stage, and microorganism tested. Although ‘Mortiño’ contained the highest phenolic and anthocyanin concentrations, it did not consistently present the lowest MIC values. Therefore, the antimicrobial response cannot be attributed solely to the total concentration of these compounds. Overall, no single fruit or ripeness stage simultaneously maximised all the physicochemical, bioactive, antioxidant, and antimicrobial characteristics evaluated. Selection of the harvest stage should consequently consider the fruit and the specific compositional or biological property of interest. Furthermore, the main limitations of this study were that the assessment of antimicrobial activity was carried out only at the M0% and M100% concentrations; therefore, further studies are required that include all stages of ripeness and identify the components responsible for the antimicrobial responses, as well as confirming the biological relevance of the antioxidant capacity measured using cell-free chemical assays.

## Figures and Tables

**Table 1:** Physicochemical characteristics of red fruits at different stages of ripeness.

Species	Maturity	Weight (g)			LD (mm)			ED (mm)			pH			SS (°Brix)			TA (%)			Humidity (%)			Ash (%)			Ca (mg/100 g DW)			Fe (mg/100 g DW)			K (mg/100 g DW)			Mg (mg/100 g DW)			Na (mg/100 g DW)		
Blackberry ‘Castilla’	M0%	2.8	±	0.6 ^c^	20.9	±	1.2 ^a^	15.8	±	1.5 ^c^	2.7	±	0.0 ^a^	6.0	±	0.0 ^c^	2.7	±	0.3 ^b^	81.6	±	1.6 ^c^	1.4	±	0.1 ^a^	1881.3	±	38.3 ^a^	6.2	+	0.8 ^a^	906.0	+	8.0 ^a^	1087.7	+	20.8 ^a^	24.6	+	1.3 ^bc^
	M50%	4.2	±	0.6 ^b^	23.0	±	0.7 ^a^	17.7	±	1.0 ^b^	2.3	±	0.1 ^c^	6.5	±	0.6 ^bc^	3.5	±	0.1 ^a^	87.2	±	0.7 ^ab^	0.5	±	0.1 ^c^	1674.9	±	14.0 ^b^	6.4	+	0.3 ^a^	718.6	+	8.9 ^b^	917.4	+	31.4 ^b^	27.0	+	4.6 ^ab^
	M80%	4.6	±	0.6 ^b^	22.0	±	1.1 ^a^	18.7	±	0.8 ^ab^	2.4	±	0.0 ^b^	7.5	±	1.7 ^b^	3.5	±	0.1 ^a^	85.7	±	3.1 ^b^	0.5	±	0.1 ^c^	1073.2	±	69.4 ^c^	5.5	+	0.6 ^b^	418.9	+	5.8 ^d^	704.2	+	55.2 ^c^	22.0	+	0.9 ^c^
	M100%	5.4	±	0.4 ^a^	21.4	±	2.2 ^a^	19.6	±	0.6 ^a^	2.5	±	0.0 ^b^	9.5	±	0.6 ^a^	2.3	±	0.1 ^c^	88.8	±	1.2 ^a^	0.8	±	0.1 ^b^	688.5	±	33.0 ^d^	5.4	+	0.2 ^b^	619.3	+	26.0 ^c^	577.6	+	5.6 ^d^	27.7	+	0.4 ^a^
Raspberry ‘Buckingham Tayberry’	M0%	1.4	±	0.4 ^c^	17.4	±	2.3 ^b^	11.5	±	2.0 ^b^	3.1	±	0.1 ^a^	6.0	±	0.0 ^d^	2.3	±	0.1 ^c^	76.9	±	2.1 ^b^	1.1	±	0.0 ^a^	715.6	±	32.6 ^a^	2.9	±	0.1 ^b^	1691.8	±	10.6 ^b^	1965.9	±	15.6 ^a^	23.6	±	5.3 ^a^
	M50%	3.1	±	0.4 ^b^	16.3	±	9.0 ^b^	11.9	±	5.8 ^b^	2.7	±	0.0 ^bc^	7.5	±	0.6 ^c^	2.6	±	0.2 ^b^	82.8	±	3.8 ^a^	0.6	±	0.0 ^b^	634.6	±	23.1 ^b^	3.1	±	0.3 ^b^	1945.7	±	6.5 ^a^	1786.5	±	4.9 ^b^	27.0	±	2.4 ^a^
	M80%	3.5	±	0.8 ^b^	22.7	±	2.7 ^ab^	15.5	±	0.8 ^ab^	2.7	±	0.0 ^b^	8.8	±	0.5 ^b^	2.9	±	0.0 ^ab^	85.0	±	0.6 ^a^	0.4	±	0.3 ^b^	523.0	±	3.9 ^c^	6.0	±	0.5 ^a^	1697.5	±	66.1 ^b^	1751.4	±	14.0 ^b^	26.4	±	3.6 ^a^
	M100%	5.2	±	1.5 ^a^	26.3	±	3.4 ^a^	17.4	±	1.6 ^a^	2.6	±	0.0 ^c^	9.8	±	1.0 ^a^	3.1	±	0.4 ^a^	85.1	±	1.0 ^a^	0.6	±	0.0 ^b^	287.9	±	2.7 ^d^	0.1	±	0.0 ^c^	1246.3	±	54.6 ^c^	1027.7	±	36.8 ^c^	24.7	±	3.9 ^a^
Raspberry ‘Heritage’	M0%	3.3	±	0.4 ^b^	19.4	±	1.3 ^a^	17.8	±	1.3 ^ab^	3.0	±	0.0 ^a^	6.9	±	0.1 ^c^	2.6	±	0.1 ^a^	87.8	±	0.7 ^a^	0.6	±	0.1 ^a^	371.7	±	26.0 ^a^	6.4	±	0.3 ^a^	1218.1	±	35.0 ^c^	1388.9	±	46.6 ^a^	26.5	±	3.5 ^a^
	M50%	3.3	±	0.3 ^b^	21.8	±	7.6 ^a^	16.8	±	0.5 ^b^	2.9	±	0.0 ^b^	8.5	±	0.6 ^b^	2.8	±	0.1 ^a^	86.4	±	0.4 ^b^	0.7	±	0.3 ^a^	299.6	±	1.7 ^b^	5.6	±	0.1 ^b^	1535.4	±	15.6 ^a^	1144.7	±	21.8 ^b^	23.0	±	0.9 ^a^
	M80%	4.1	±	0.5 ^a^	20.2	±	1.2 ^a^	18.6	±	0.9 ^a^	2.9	±	0.1 ^b^	9.3	±	0.5 ^a^	2.5	±	0.1 ^a^	86.8	±	0.5 ^b^	0.6	±	0.4 ^a^	280.0	±	39.5 ^b^	3.0	±	0.5 ^c^	1427.8	±	12.6 ^b^	1054.7	±	11.1 ^abc^	23.7	±	7.0 ^a^
	M100%	3.8	±	0.4 ^ab^	19.2	±	1.9 ^a^	18.2	±	1.0 ^ab^	2.9	±	0.0 ^b^	8.8	±	0.5 ^ab^	1.7	±	0.3 ^b^	88.5	±	0.4 ^a^	0.5	±	0.1 ^a^	291.1	±	38.8 ^b^	0.1	±	0.0 ^d^	1234.5	±	64.8 ^c^	916.1	±	11.3 ^c^	28.9	±	5.5 ^a^
Blueberry	M0%	1.4	±	0.1 ^c^	11.1	±	1.2 ^a^	14.8	±	0.3 ^a^	2.6	±	0.2 ^ab^	8.3	±	0.5 ^c^	3.3	±	0.1 ^a^	86.2	±	2.2 ^a^	0.3	±	0.0 ^a^	279.6	±	10.1 ^c^	0.1	±	0.0 ^b^	482.0	±	15.8 ^a^	1560.7	±	46.6 ^a^	36.1	±	2.4 ^ab^
	M50%	1.8	±	0.3 ^bc^	9.4	±	0.5 ^b^	15.1	±	0.8 ^a^	2.5	±	0.0 ^b^	10.5	±	0.6 ^b^	1.9	±	0.1 ^b^	86.2	±	0.6 ^a^	0.3	±	0.0 ^ab^	885.3	±	47.7 ^ab^	0.1	±	0.0 ^b^	187.6	±	3.4 ^c^	705.2	±	6.8 ^b^	29.7	±	2.4 ^b^
	M80%	2.5	±	0.6 ^a^	9.6	±	1.0 ^b^	16.3	±	2.0 ^a^	2.6	±	0.0 ^ab^	12.3	±	0.5 ^a^	1.9	±	0.1 ^b^	81.6	±	2.5 ^b^	0.2	±	0.0 ^b^	919.5	±	76.9 ^a^	5.4	±	1.2 ^a^	165.4	±	8.6 ^d^	352.8	±	8.6 ^c^	12.9	±	1.7 ^c^
	M100%	2.1	±	0.2 ^ab^	10.1	±	0.5 ^ab^	15.7	±	1.0 ^a^	2.7	±	0.0 ^a^	11.8	±	0.5 ^a^	1.9	±	0.1 ^b^	85.8	±	1.7 ^a^	0.3	±	0.0 ^a^	828.0	±	10.4 ^b^	0.1	±	0.0 ^b^	217.4	±	3.0 ^b^	370.5	±	6.5 ^c^	39.2	±	1.2 ^a^
‘Mortiño’	M0%	0.1	±	0.0 ^b^	5.9	±	0.4 ^a^	5.8	±	0.1 ^ab^	2.8	±	0.0 ^a^	8.7	±	1.3 ^b^	1.7	±	0.2 ^a^	79.1	±	1.2 ^c^	0.5	±	0.2 ^a^	372.5	±	12.9 ^a^	0.1	±	0.0 ^b^	829.8	±	11.3 ^a^	576.1	±	60.7 ^a^	24.1	±	6.7 ^ab^
	M50%	0.1	±	0.0 ^b^	5.4	±	0.2 ^a^	5.8	±	0.4 ^b^	2.8	±	0.0 ^a^	7.9	±	1.7 ^b^	1.4	±	0.1 ^b^	79.4	±	0.7 ^c^	0.5	±	0.1 ^a^	400.4	±	28.1 ^a^	3.1	±	0.4 ^a^	777.1	±	37.3 ^b^	538.9	±	54.7 ^ab^	32.3	±	8.5 ^a^
	M80%	0.2	±	0.0 ^b^	5.8	±	0.4 ^a^	6.4	±	0.3 ^a^	2.7	±	0.0 ^b^	8.9	±	0.2 ^b^	1.7	±	0.0 ^a^	81.7	±	1.0 ^b^	0.5	±	0.3 ^a^	314.8	±	52.9 ^b^	0.1	±	0.0 ^b^	714.7	±	1.3 ^c^	508.3	±	57.1 ^b^	28.5	±	7.8 ^ab^
	M100%	0.7	±	0.1 ^a^	2.1	±	0.5 ^b^	1.9	±	0.6 ^c^	2.7	±	0.0 ^b^	12.4	±	0.8 ^a^	1.0	±	0.1 ^c^	84.3	±	1.1 ^a^	0.1	±	0.0 ^b^	235.0	±	7.5 ^c^	3.1	±	0.0 ^a^	724.2	±	37.6 ^c^	397.1	±	3.8 ^c^	21.6	±	0.2 ^b^
A_M0%_		***			***			***			***			***			***			***			***			***			***			***			***			***		
A_M50%_		***			***			***			***			***			***			***			**			***			***			***			***			*		
A_M80%_		***			***			***			***			***			***			**			ns			***			***			***			***			***		
A_M100%_		***			***			***			***			***			***			***			***			***			***			***			***			**		

**Note:** Values are expressed as the average ± standard deviation. LD, longitudinal diameter; ED, equatorial diameter; SS, soluble solids; TA, titratable acidity; Ca, calcium; Fe, iron; K, potassium; Mg, magnesium; Na, sodium. The lowercase letters next to the deviation indicate significant differences between different degrees of ripeness for the same fruit. A_M0%_ indicates significant differences in the same degree of ripeness (M0%) across all fruits in the study. ns, not significant; *, *p* < 0.05; **, *p* < 0.01; *** *p* < 0.001.

**Table 2:** Bioactive compound concentration and antioxidant activity in red fruits at different stages of ripeness.

Species	Maturity	Vitamin C (mg/100 g DW)			Citric Acid (mg/100 g DW)			Malic Acid (mg/100 g DW)			Tartaric Acid (mg/100 g DW)			Total Organic Acid (mg/100 g DW)			Antioxidant Activity (mmol ET/100 g DW)					
																	DPPH			ABTS		
Blackberry ‘Castilla’	M0%	10.9	±	0.4 ^b^	2041.0	±	7.0 ^c^	313.6	±	85.1 ^a^	328.7	±	63.1 ^b^	2683.3	±	63.1 ^c^	5.8	±	0.1 ^a^	6.8	±	0.2 ^a^
	M50%	7.8	±	0.1 ^c^	2847.4	±	18.9 ^b^	190.5	±	15.6 ^b^	362.7	±	47.3 ^a^	3400.7	±	47.3 ^a^	5.3	±	0.7 ^ab^	6.7	±	0.2 ^a^
	M80%	5.6	±	0.3 ^d^	2802.9	±	167.5 ^b^	75.6	±	17.8 ^c^	268.9	±	51.0 ^c^	3147.4	±	51.0 ^b^	5.2	±	0.2 ^b^	6.2	±	0.1 ^ab^
	M100%	37.9	±	0.6 ^a^	3172.1	±	120.5 ^a^	211.2	±	24.0 ^b^	187.9	±	20.1 ^d^	3571.3	±	20.1 ^a^	5.3	±	0.7 ^ab^	5.6	±	0.4 ^b^
Raspberry ‘Buckingham Tayberry’	M0%	9.1	±	0.2 ^d^	2416.7	±	184.5 ^c^	2375.7	±	15.4 ^a^	225.6	±	15.9 ^c^	5018.0	±	15.9 ^c^	4.9	±	0.2 ^b^	6.1	±	0.3 ^b^
	M50%	30.9	±	2.0 ^a^	4353.8	±	224.6 ^a^	1956.0	±	95.4 ^b^	303.3	±	15.5 ^a^	6613.1	±	15.5 ^a^	5.6	±	0.1 ^ab^	6.6	±	0.1 ^a^
	M80%	16.4	±	1.5 ^c^	4211.9	±	42.2 ^a^	1513.7	±	9.2 ^c^	250.1	±	3.8 ^b^	5975.7	±	3.8 ^b^	5.7	±	0.1 ^a^	4.4	±	0.3 ^c^
	M100%	24.4	±	1.1 ^b^	3009.3	±	92.0 ^b^	832.5	±	0.3 ^d^	142.8	±	6.2 ^d^	3984.6	±	6.2 ^d^	6.1	±	0.5 ^a^	6.8	±	0.1 ^a^
Raspberry ‘Heritage’	M0%	15.3	±	1.2 ^c^	2845.2	±	5.9 ^c^	310.7	±	5.4 ^a^	67.5	±	6.2 ^b^	3223.4	±	6.2 ^c^	5.9	±	0.91 ^a^	6.5	±	0.1 ^a^
	M50%	54.4	±	2.6 ^a^	3733.3	±	62.1 ^a^	274.2	±	32.1 ^b^	101.8	±	7.7 ^a^	4109.3	±	7.7 ^a^	5.2	±	0.56 ^c^	5.4	±	0.5 ^b^
	M80%	56.9	±	4.4 ^a^	3283.3	±	128.3 ^b^	243.9	±	3.3 ^c^	44.7	±	1.1 ^c^	3571.9	±	1.1 ^b^	5.0	±	0.88 ^d^	6.1	±	0.5 ^ab^
	M100%	44.5	±	0.9 ^b^	2447.7	±	53.0 ^d^	184.5	±	0.9 ^d^	11.0	±	0.0 ^d^	2643.2	±	0.0 ^d^	5.4	±	0.96 ^b^	5.6	±	0.8 ^b^
Blueberry	M0%	nd			2133.3	±	179.6 ^a^	2637.5	±	370.2 ^a^	302.6	±	23.0 ^a^	5073.5	±	23.0 ^a^	5.3	±	0.1 ^b^	6.5	±	0.4 ^a^
	M50%	nd			2254.8	±	117.5 ^a^	1779.6	±	98.7 ^b^	71.9	±	3.8 ^c^	4106.4	±	3.8 ^b^	5.8	±	0.4 ^ab^	6.4	±	0.2 ^a^
	M80%	nd			1638.3	±	129.9 ^b^	1116.3	±	72.2 ^c^	135.9	±	8.8 ^b^	2890.5	±	8.8 ^c^	5.4	±	0.9 ^ab^	6.5	±	0.1 ^a^
	M100%	2.7	±	0.13	392.0	±	2.7 ^c^	207.7	±	39.8 ^d^	23.3	±	9.3 ^d^	668.9	±	9.3 ^d^	6.0	±	0.2 ^a^	6.5	±	0.2 ^a^
‘Mortiño’	M0%	0.8	±	0.0 ^c^	314.2	±	2.6 ^b^	1471.1	±	32.7 ^b^	940.7	±	16.1 a	2803.9	±	16.1 ^b^	6.3	±	0.2 ^a^	5.5	±	0.0 ^a^
	M50%	0.7	±	0.0 ^c^	383.4	±	2.9 ^c^	1432.2	±	14.7 ^b^	786.8	±	16.2 ^b^	2533.1	±	16.2 ^b^	5.4	±	0.6 ^b^	5.5	±	0.1 ^a^
	M80%	4.8	±	0.2 ^b^	480.5	±	3.8 ^b^	2281.4	±	1.0 ^a^	599.9	±	11.1 ^c^	3264.8	±	11.1 ^a^	4.9	±	0.7 ^a^	5.4	±	0.6 ^a^
	M100%	5.2	±	0.2 ^a^	392.0	±	2.7 ^a^	2387.5	±	12.6 ^a^	317.1	±	3.5 ^d^	3182.1	±	3.5 ^a^	5.9	±	0.9 ^a^	5.8	±	0.1 ^a^
A_M0%_		***			***			***			***			***			**			**		
A_M50%_		***			***			***			***			***			*			***		
A_M80%_		***			***			***			***			***			ns			***		
A_M100%_		***			***			***			***			***			**			*		

**Note:** Values are expressed as the average ± standard deviation of the major compounds. nd, undetectable. The lowercase letters next to the deviation indicate significant differences between different degrees of ripeness for the same fruit. A_M0%_ indicates significant differences in the same degree of ripeness (M0%) across all fruits in the study. ns, not significant; *, *p* < 0.05; **, *p* < 0.01; *** *p* < 0.001.

**Table 3:** Carotenoid profile of fruits at different stages of ripeness.

Species	Maturity	α-Carotene (mg/100 g DW)			β-Carotene (mg/100 g DW)			Lutein (mg/100 g DW)			9-Cis Anteraxanthina (mg/100 g DW)			Zeaxanthin (mg/100 g DW)			Zeinoxanthin (mg/100 g DW)			Total Carotenoids (mg/100 g DW)			Pheophytin a (mg/100 g DW)			Pheophytin b (mg/100 g DW)			Chlorophyll b (mg/100 g DW)			Total Chlorophyll and Derivatives (mg/100 g DW)		
Blackberry ‘Castilla’	M0%							3.2	±	0.3 ^a^										3.2	±	0.3 ^a^				27.3	±	1.6 ^a^	10.2	±	0.8	37.5	±	2.4 ^a^
	M50%							1.0	±	0.0 ^b^										1.0	±	0.0 ^b^				10.5	±	0.1 ^b^				10.5	±	0.1 ^b^
	M80%							0.3	±	0.0 ^c^										0.2	±	0.0 ^c^				2.7	±	0.0 ^c^				2.7	±	0.0 ^c^
	M100%							0.2	±	0.0 ^c^										0.2	±	0.0 ^c^				0.9	±	0.0 ^d^				0.9	±	0.0 ^d^
Raspberry ‘Buckingham Tayberry’	M0%							1.9	±	0.1 ^a^				0.2	±	0.0 ^a^				2.1	±	0.1 ^a^				21.5	±	3.3 ^a^	3.1	±	0.7	24.5	±	4.0 ^a^
	M50%							0.6	±	0.1 ^b^							0.1	±	0.0 ^a^	0.6	±	0.1 ^c^				5.1	±	0.2 ^b^				5.1	±	0.2 ^b^
	M80%							0.4	±	0.0 ^c^	1.1	±	0.1	0.1	±	0.0 ^b^	0.1	±	0.0 ^a^	1.5	±	0.1 ^b^	0.7	±	0.0	4.6	±	0.1 ^b^				5.3	±	0.1 ^b^
	M100%							0.3	±	0.1 ^d^							0.1	±	0.0 ^a^	0.3	±	0.1 ^d^				1.4	±	0.2 ^c^				1.4	±	0.2 ^c^
Raspberry ‘Heritage’	M0%				0.4	±	0.0 ^c^	0.4	±	0.1 ^d^				0.1	±	0.0 ^c^	0.1	±	0.0 ^c^	0.8	±	0.1 ^c^				0.7	±	0.0 ^b^				0.7	±	0.0
	M50%				9.0	±	0.8 ^a^	2.4	±	0.0 ^a^				0.2	±	0.0 ^a^	0.1	±	0.0 ^a^	11.6	±	0.7 ^a^				1.0	±	0.0 ^a^						
	M80%	0.8	±	0.1 ^b^	6.1	±	0.0 ^b^	1.4	±	0.1 ^b^				0.1	±	0.0 ^b^	0.1	±	0.0 ^a^	8.5	±	0.0 ^b^												
	M100%	1.0	±	0.2 ^a^	9.1	±	0.9 ^a^	0.9	±	0.0 ^c^							0.1	±	0.0 ^b^	11.1	±	1.1 ^a^												
Blueberry	M0%							0.6	±	0.0 ^c^							0.2	±	0.0	0.7	±	0.0 ^c^				26.1	±	1.0 ^c^				26.1	±	1.0 ^c^
	M50%							0.3	±	0.0 ^d^										0.2	±	0.0 ^d^	8.4	±	0.1 ^a^	9.3	±	1.5 ^d^				22.2	±	1.6 ^c^
	M80%							2.6	±	0.0 ^b^	1.2	±	0.0 ^b^							4.0	±	0.3 ^b^	5.3	±	0.8 ^c^	35.9	±	3.4 ^b^				39.1	±	4.2 ^b^
	M100%							4.0	±	0.4 ^a^	3.0	±	0.0 ^a^							7.2	±	0.7 ^a^	7.1	±	0.5 ^b^	59.8	±	4.8 ^a^				67.3	±	5.3 ^a^
‘Mortiño’	M0%							2.5	±	0.3 ^a^	1.0	±	0.1 ^a^	0.1	±	0.0 ^c^				3.6	±	0.3 ^a^				13.9	±	1.3 ^a^	4.7	±	0.5	18.6	±	1.8 ^a^
	M50%							0.7	±	0.3 ^b^	0.5	±	0.1 ^b^	0.1	±	0.1 ^c^				1.3	±	0.3 ^b^				6.2	±	0.8 ^b^				6.2	±	0.8 ^b^
	M80%							0.1	±	0.1 ^c^	0.1	±	0.1 ^c^							0.2	±	0.0 ^c^				0.7	±	0.1 ^c^				0.7	±	0.1 ^c^
	M100%							0.1	±	0.0 ^c^	0.1	±	0.0 ^c^							0.2	±	0.0 ^c^				0.2	±	0.0 ^d^				0.2	±	0.0 ^d^
A_M0%_								***						***			***			***						***			***			***		
A_M50%_								***						**			*			***						***						***		
A_M80%_								***			***			***						***			***			***						***		
A_M100%_								***			***						ns			***						***						***		

**Note:** Values are expressed as the average ± standard deviation of the major compounds. The lowercase letters next to the deviation indicate significant differences between different degrees of ripeness for the same fruit. A_M0%_ indicates significant differences in the same degree of ripeness (M0%) across all fruits in the study. ns, not significant; *, *p* < 0.05; **, *p* < 0.01; *** *p* < 0.001.

**Table 4:** Phenolic profile of fruits at different stages of ripeness.

Species	Maturity	Gallic Acid (mg/100 g DW)			4-Hydroxybenzoic acid (mg/100 g DW)			*m*-Coumaric Acid (mg/100 g DW)			Syringic Acid (mg/100 g DW)			Chlorogenic Acid (mg/100 g DW)			Caffeic Acid (mg/100 g DW)			Ferulic Acid (mg/100 g DW)			Kaempferol (mg/100 g DW)			Quercetin Glucoside (mg/100 g DW)			Quercetin (mg/100 g DW)			Total Phenolics (mg/100 g DW)			Total Anthocyanins (mg/100 g DW)		
Blackberry ‘Castilla’	M0%	5.4	±	0.6 ^a^							12.2	±	0.9 ^c^	12.7	±	0.6 ^d^	184.9	±	10.1 ^a^	119.1	±	4.4 ^a^	15.0	±	0.5 ^a^	23.3	±	0.4 ^a^	49.3	±	1.7 ^a^	421.9	±	10.6 ^c^	23.7	±	3.4 ^c^
	M50%	2.2	±	0.4 ^c^							67.5	±	5.2 ^a^	59.2	±	7.3 ^b^	167.3	±	5.9 ^b^	95.0	±	6.9 ^b^	9.1	±	0.3 ^b^	17.8	±	0.4 ^c^	44.2	±	4.1 ^b^	462.4	±	6.2 ^b^	40.0	±	0.9 ^b^
	M80%	3.7	±	0.4 ^b^							54.2	±	0.1 ^b^	66.3	±	0.1 ^a^	133.2	±	1.0 ^c^	98.7	±	0.3 ^b^	2.1	±	0.0 ^d^	7.9	±	0.1 ^d^	21.7	±	0.4 ^d^	388.0	±	1.1 ^d^	40.2	±	9.9 ^b^
	M100%	4.9	±	0.5 ^a^				483.0	±	30.0	12.8	±	0.3 ^c^	47.3	±	2.1 ^c^	67.5	±	7.0 ^d^	37.2	±	2.3 ^c^	5.5	±	0.5 ^c^	20.0	±	1.2 ^b^	31.6	±	1.7 ^c^	709.9	±	45.7 ^a^	231.2	±	15.9 ^a^
Raspberry ‘Buckingham Tayberry’	M0%	7.5	±	1.0 ^a^	33.5	±	0.9 ^d^	9.3	±	0.7 ^b^	6.4	±	0.0 ^b^	23.3	±	0.8 ^d^	32.5	±	1.2 ^b^							7.5	±	0.1 ^bc^	10.8	±	0.4 ^b^	130.7	±	0.1 ^d^	24.2	±	3.7 ^b^
	M50%	5.6	±	0.6 ^b^	70.8	±	0.1 ^b^	5.8	±	0.0 ^c^	5.0	±	0.0 ^c^	36.4	±	0.0 ^c^	34.6	±	6.9 ^b^							6.7	±	1.5 ^c^	14.0	±	1.1 ^a^	178.9	±	10.2 ^c^	18.1	±	0.6 ^b^
	M80%	7.1	±	0.2 ^a^	78.4	±	7.5 ^a^	13.4	±	0.4 ^a^	5.1	±	0.6 ^c^	169.4	±	7.3 ^b^	77.4	±	9.4 ^a^							8.3	±	0.4 ^b^	13.1	±	0.9 ^a^	372.2	±	3.9 ^b^	77.5	±	36.3 ^a^
	M100%	3.0	±	0.2 ^c^	59.6	±	2.6 ^c^	5.7	±	0.0 ^c^	8.6	±	0.0 ^a^	516.3	±	17.9 ^a^	28.5	±	6.8 ^b^				6.7	±	0.6	11.4	±	0.4 ^a^	6.7	±	0.0 ^c^	646.5	±	26.4 ^a^	113.6	±	31.8 ^a^
Raspberry ‘Heritage’	M0%	6.4	±	1.3 ^a^	60.0	±	0.2 ^d^	10.4	±	0.2 ^d^	5.8	±	0.0 ^c^													nd			9.1	±	1.3 ^c^	91.7	±	2.3 ^d^	21.9	±	2.5 ^d^
	M50%	6.4	±	0.6 ^a^	123.5	±	16.7 ^a^	53.4	±	8.7 ^c^	9.1	±	1.7 ^a^													2.7	±	0.5 ^a^	14.7	±	1.2 ^a^	209.8	±	29.5 ^c^	62.9	±	3.5 ^c^
	M80%	4.8	±	0.5 ^b^	110.0	±	0.3 ^b^	196.2	±	4.3 ^b^	7.3	±	0.2 ^b^										3.3	±	0.2 ^a^	2.8	±	0.5 ^a^	11.6	±	0.8 ^b^	335.9	±	5.2 ^b^	6.1	±	15.3 ^b^
	M100%	6.7	±	0.3 ^a^	91.7	±	0.6 ^c^	273.7	±	7.1 ^a^	6.3	±	0.4 ^bc^										3.4	±	0.0 ^a^	2.2	±	0.3 ^a^	8.8	±	0.2 ^c^	392.8	±	7.3 ^a^	112.4	±	4.7 ^a^
Blueberry	M0%	5.5	±	0.5 ^a^							3.7	±	0.2 ^c^	1925.0	±	54.7 ^a^	896.7	±	41.7 ^a^	454.7	±	7.5 ^a^	22.2	±	2.2 ^a^	70.8	±	6.6 ^a^	74.4	±	0.7 ^a^	3453.0	±	11.8 ^a^	8.4	±	1.8 ^c^
	M50%	4.0	±	0.2 ^b^							5.5	±	0.4 ^b^	953.7	±	2.1 ^b^	237.1	±	7.8 ^b^	296.8	±	29.7 ^c^	14.0	±	0.8 ^c^	45.8	±	2.7 ^c^	37.2	±	2.3 ^b^	1594.1	±	41.8 ^b^	22.3	±	3.7 ^c^
	M80%	3.4	±	0.0 ^c^							7.5	±	0.3 ^a^	880.9	±	21.0 ^c^	174.2	±	0.8 ^c^	352.7	±	20.9 ^b^	18.3	±	1.9 ^b^	61.2	±	1.0 ^b^	36.1	±	0.5 ^b^	1534.3	±	2.3 ^b^	75.4	±	15.9 ^b^
	M100%	2.0	±	0.0 ^d^										573.6	±	18.6 ^d^	30.0	±	1.5 ^d^	253.1	±	7.7 ^d^	4.3	±	0.9 ^d^	43.2	±	2.8 ^c^	28.1	±	0.8 ^c^	934.3	±	29.4 ^c^	512.9	±	49.2 ^a^
‘Mortiño’	M0%	28.5	±	1.6 ^a^										106.9	±	10.3 ^a^	2399.0	±	186.7 ^a^	948.4	±	10.8 ^b^	80.0	±	16.9 ^b^	63.9	±	64.7 ^a^	83.6	±	4.3 ^b^	3710.3	±	286.7 ^b^	13.9	±	0.7 ^c^
	M50%	26.2	±	0.7 ^b^										74.8	±	5.6 ^b^	2020.3	±	18.5 ^b^	2645.6	±	128.6 ^a^	110.5	±	8.9 ^a^	6.4	±	1.2 ^b^	121.4	±	0.9 ^a^	5005.2	±	140.9 ^a^	33.1	±	1.9 ^b^
	M80%	16.7	±	0.5 ^c^										13.0	±	1.2 ^d^	1039.2	±	18.3 ^d^	1031.7	±	18.6 ^b^	20.3	±	2.7 ^d^	2.8	±	0.5 ^d^	114.3	±	1.5 ^a^	2237.9	±	31.6 ^c^	30.9	±	1.7 ^b^
	M100%	9.5	±	2.3 ^d^										54.2	±	4.5 ^c^	1472.7	±	39.7 ^c^	318.2	±	37.2 ^c^	60.9	±	2.7 ^c^	10.0	±	0.6 ^c^	92.9	±	14.9 ^b^	2018.4	±	45.9 ^c^	970.7	±	46.9 ^a^
A_M0%_		***			***			**			***			***			***			***			***			*			***			***			***		
A_M50%_		***			***			***			***			***			***			***			***			***			***			***			***		
A_M80%_		***			***			***			***			***			***			***			***			***			***			***			**		
A_M100%_		***			***			***			***			***			***			***			***			***			***			***			***		

**Note:** Values are expressed as the average ± standard deviation of the major compounds. nd, undetectable. The lowercase letters next to the deviation indicate significant differences between different degrees of ripeness for the same fruit. A_M0%_ indicates significant differences in the same degree of ripeness (M0%) across all fruits in the study. *, *p* < 0.05; **, *p* < 0.01; *** *p* < 0.001.

**Table 5:** Minimum inhibitory concentration of dry ethanolic extracts of red fruits against different microorganisms.

	Minimum Inhibitory Concentration (mg/mL)									
Microorganisms	Blackberry ‘Castilla’		Raspberry ‘Buckingham Tayberry ‘		Raspberry ‘Heritage’		Blueberry		‘Mortiño’	
	M0%	M100%	M0%	M100%	M0%	M100%	M0%	M100%	M0%	M100%
Bacteria										
*Cutibacterium acnes* ATCC 11827	2.3	1.2	3.5	7.0	7.0	14.1	7.0	11.8	2.3	3.5
*Edwardsiella tarda* ATCC 15947	18.8	18.8	9.5	9.5	18.8	38.2	18.8	75.7	9.5	37.6
*Enterococcus faecalis* ATCC 49533	9.4	37.5	14.1	9.4	7.0	9.4	9.4	18.8	9.4	37.5
*Escherichia coli* ATCC 8739	37.5	75.0	18.8	37.5	18.8	37.5	37.5	18.8	37.5	18.8
*Klebsiella pneumoniae* ATCC 10031	37.5	18.8	2.3	9.4	9.4	9.4	9.4	37.5	18.8	56.3
*Listeria. monocitogenes* ATCC 19114	3.5	9.4	3.5	28.1	9.4	18.8	9.4	28.1	4.7	18.8
*Porphyromonas gingivalis* ATCC 33277	3.5	9.4	112.5	7.0	9.4	18.8	9.4	14.1	9.4	18.8
*Pseudomonas aeruginosa* ATCC 9027	9.4	37.5	18.8	37.5	18.8	37.5	37.5	75.0	18.8	37.5
*Pseudomonas fluorescens* ATCC 13525	14.1	18.8	18.9	14.2	14.1	19.1	9.4	151.5	9.5	37.6
*Pseudomonas putida* ATCC 31483	14.1	9.4	9.5	9.5	9.4	19.1	9.4	75.7	9.5	37.6
*Staphylococcus aureus* ATCC 6538P	9.4	18.4	18.8	37.5	18.8	37.5	18.8	37.5	18.8	75.0
*Staphylococcus epidermidis* ATCC 12228	9.4	14.1	4.7	18.9	9.4	14.4	14.1	75.7	4.7	37.6
*Streptococcus mutans* ATCC 25175	9.4	9.4	18.8	18.8	9.4	18.8	9.4	9.4	1.2	37.5
Fungi										
*Aspergillus niger* ATCC 6275	-	-	-	-	-	-	-	-	-	-
*Candida albicans* ATCC 10231	18.8	37.5	-	-	-	-	35.0	150.0	150.0	75.0
*Candida tropicalis* ATCC 13803	75.0	150.0	-	-	-	-	75.0	150.0	150.0	75.0
*Fusarium keratoplasticum* ATCC 36031	18.8	75.0	37.5	150.0	56.3	112.5	37.5	-	56.3	112.5
*Malassezia furfur* ATCC 14521	1.2	1.2	1.2	2.3	1.2	1.2	1.2	1.2	1.2	1.2
*Microsporum canis* ATCC 36299	7.0	4.7	2.3	2.3	4.7	9.4	2.3	28.1	3.5	9.4
*Trichophyton interdigitale* ATCC 9533	2.3	2.3	9.4	7.0	3.5	3.5	7.0	28.1	4.7	3.5

**Note:** -, not active at the tested concentration. Some values are arithmetic means of duplicate inhibitory endpoints. Therefore, mean values between two dilution steps are summary statistics rather than additional tested concentrations.

## Data Availability

The data supporting the findings of this study are available on request from the corresponding author.
